# Transcription Activator-Like Effector Nuclease (TALEN)-Mediated *CLYBL* Targeting Enables Enhanced Transgene Expression and One-Step Generation of Dual Reporter Human Induced Pluripotent Stem Cell (iPSC) and Neural Stem Cell (NSC) Lines

**DOI:** 10.1371/journal.pone.0116032

**Published:** 2015-01-14

**Authors:** Trevor Cerbini, Ray Funahashi, Yongquan Luo, Chengyu Liu, Kyeyoon Park, Mahendra Rao, Nasir Malik, Jizhong Zou

**Affiliations:** 1 NIH Center for Regenerative Medicine, Laboratory of Stem Cell Biology, National Institute of Arthritis, Musculoskeletal and Skin Diseases, Bethesda, Maryland, United States of America; 2 Center for Molecular Medicine, Division of Intramural Research, National Heart, Lung, and Blood Institute, Bethesda, Maryland, United States of America; 3 Stem Cell Unit, National Institute of Neurological Disorders and Stroke, Bethesda, Maryland, United States of America; Johns Hopkins School of Medicine, UNITED STATES

## Abstract

Targeted genome engineering to robustly express transgenes is an essential methodology for stem cell-based research and therapy. Although designer nucleases have been used to drastically enhance gene editing efficiency, targeted addition and stable expression of transgenes to date is limited at single gene/locus and mostly *PPP1R12C/AAVS1* in human stem cells. Here we constructed transcription activator-like effector nucleases (TALENs) targeting the safe-harbor like gene *CLYBL* to mediate reporter gene integration at 38%–58% efficiency, and used both AAVS1-TALENs and CLYBL-TALENs to simultaneously knock-in multiple reporter genes at dual safe-harbor loci in human induced pluripotent stem cells (iPSCs) and neural stem cells (NSCs). The CLYBL-TALEN engineered cell lines maintained robust reporter expression during self-renewal and differentiation, and revealed that *CLYBL* targeting resulted in stronger transgene expression and less perturbation on local gene expression than *PPP1R12C/AAVS1*. TALEN-mediated *CLYBL* engineering provides improved transgene expression and options for multiple genetic modification in human stem cells.

## Introduction

Human pluripotent and multipotent stem cells are important platforms for studying human development and disease mechanisms, and are promising resources for stem cell based drug-screening, cell replacement, and gene therapies, because of their self-renewal properties and differentiation potentials. Compared to work done in mouse pluripotent stem cells and human cancer cell lines, genome engineering in human pluripotent stem cells has been challenging partially due to low transfection/transduction efficiency and high apoptosis under stresses such as low-density plating, drug-selection and sorting. Besides improvements in the delivery of nucleotides and cell culture conditions, quantum leaps of genetic modification efficiencies were achieved through the application of designer nucleases, including zinc finger nucleases (ZFNs), transcription activator-like effector nuclease (TALEN) and clustered regularly interspaced short palindromic repeat (CRISPR) RNA-guided Cas nuclease (CRISPR/Cas) in the last decade [1]. We and others have shown efficient gene targeting in human embryonic stem cells (ESCs) and induced pluripotent stem cells (iPSCs) using these designer nucleases [2, 3, 4]. Aided by fluorescence activated cell sorting (FACS) of transfected cells, TALENs and CRISPR/Cas were shown to mediate high-efficiency single-gene indel mutagenesis through the error-prone non-homologous end-joining (NHEJ) mechanism and defined single-nucleotide alterations through homology-directed repair (HDR) mechanism in human pluripotent stem cells [5, 6]. However, applications of human stem cells in imaging, drug-screening and gene therapy would prefer using cells bearing targeted insertion and stable expression of large DNA fragments such as reporters or minigene cassettes [7, 8]. Therefore it’s also highly desirable to engineer multiple genes simultaneously in human pluripotent stem cells to save time and cost. The one-step generation of mouse ESCs and mice carrying multiple indels [9], and rat with multiple floxed alleles [10] indicated it’s possible to do so with highly active designer nucleases.

Safe-harbor loci, which permit robust expression of integrated transgenes in the mammalian genome, provide defined “landing sites” for large exogenous DNA, such as minigenes and reporter cassettes. Ideally, genomic safe-harbors are loci that not only enable adequate, stable expression of the integrated material, but also minimize impacts on any nearby endogenous gene structures or functions. Focusing on the safety concerns, one group has proposed criteria by which to evaluate potential safe-harbors based on known human genome information, including; a distance of at least 50kb from the 5’ end of any gene, at least 300kb from any cancer-related gene and microRNA, and a location outside of transcriptional units and ultraconserved regions [11]. While the proposed set of guidelines is helpful to identify potential safe-harbors, if strictly adhered to, it would exclude some widely studied and used safe-harbors, such as *PPP1R12C/AAVS1 and hROSA26,*which have been shown to allow robust transgene expression in engineered human pluripotent stem cells and their differentiated lineages [3, 8, 12]. Therefore we have taken a different approach to identify a genomic safe-harbor that enables sustainable gene expression and can be efficiently and definitively targeted by designer nucleases to engineer various human cell types.

In this paper we describe a novel target for TALEN–enhanced integrative gene-transfer, located in intron 2 of the Citrate Lyase Beta-Like (CLYBL) gene, on the long arm of chromosome 13. CLYBL was one of the identified random integration hot spots of the phage-derived phiC31 integrase, and a subsequent study detailed the stability of randomly integrated transgenes expressed from this locus [13, 14]. Using highly sensitive and quantitative reporter genes contained in the targeting donors, we targeted constructs to both the *CLBYL*and AAVS1 safe harbors and surprisingly found that the CLYBL safe-harbor enables up to 10-fold higher transgene expression than AAVS1. High-efficiency CLYBL-TALENs and AAVS1-TALENs also enabled dual reporter knock-in at dual safe-harbors simultaneously, in both human iPSCs and NSCs, without perturbation of differentiation potentials or gene expression, either globally or locally. Establishing and characterizing the CLYBL safe-harbor locus opens up greater potential in genome engineering applications, such as the generation of more developmentally-relevant pluripotent stem cell reporter lines and multi-gene therapy interventions.

## Materials and Methods

### Human iPSCs and NSCs culture

Human NCRM5 iPSCs [15] were maintained on hESC-qualified Matrigel Basement Membrane Matrix (BD #354277) and cultured with Essential 8 Medium (Invitrogen #A14666SA) as per each manufacturer’s instructions. Media was refreshed daily. For passaging, dissociation buffer was made by adding 500μl 0.5M EDTA and 0.9g NaCl into 500ml of Calcium and Magnesium free PBS (Invitrogen #14190). Cells were routinely passaged at 80% confluence. Low passage (<20 passages) H9 ESC and NCRM1 iPSC derived human NSCs were purchased from Life Technologies (N7800–100) and XCell through a service contract, respectively, and cultured in 37^o^C tissue culture incubator with StemPro NSC Serum Free Medium (SFM) (Life Technologies #A10509–01) on plates coated with Geltrex LDEV-Free Reduced Growth Factor Basement Membrane Matrix (Life Technologies #A1413201) and passaged by StemPro Accustase (Invitrogen #A1110501).

### Gene targeting in human iPSCs and NSCs

Human iPSCs were passaged two days before transfection using StemPro Accustase at an appropriate density to achieve roughly 80% confluency in 48 hours. For transfection, 3×10^6^ cells were harvested with Accutase. Cells were resuspended in 100μl P3 Primary Cell 4D-Nucleofector X Solution (Lonza # V4XP-3024) with 5μg each TALEN and 10μg donor plasmid and transfected using the 4D-Nucleofector X Unit (Lonza #AAF-1001X) and preset program CB-150. Cells were replated onto DR4 MEFs (GlobalStem #GSC-6004G-C) in 3 wells of a 6-well plate and E8 media was supplemented with 10 μM ROCK inhibitor Y27632 for 24 hours post nucleofection. Nuleofection of human NSCs was carried out using 4D-Nucleofector X Unit with P4 Primary Cell X Kit L (Lonza #V4XP-4012) and program DN100. Prior to nucleofection, 3.5 million NSCs were detached with StemPro Accutase and harvested by centrifugation at 1000rpm for 4 min. The supernatant was removed and the cell pellets were resuspended in 100μl of P4 solution. Left TALEN, right TALEN, and donor vector DNA were added in a 1:1:1 ratio. Total DNA:cell ratio was 2ug/million NSCs. After nucleofection, 500ul of pre-warmed StemPro NSC SFM was added to the cells and total solution was immediately transferred to a single well of a Geltrex coated 6-well plate filled with 2ml of pre-warmed StemPro NSC SFM.

### Drug selection of targeted human iPSCs and NSCs

For human iPSC selection, puromycin or G418 concentration was first optimized in untargeted human iPSCs by a kill-curve analysis. At 2–3 days post nucleofection, NutriStem XF/FF medium (Stemgent #01–0005) supplemented with either 0.25μg/ml puromycin or 25μg/ml G418 was used to replace E8 medium and refreshed every day for up to 7–12 days or until selection appeared complete (when untargeted control cells were all killed). For dual safe-harbor targeting, cells were first subjected to puromycin selection for 7 days, after which G418 selection was performed for 7 days before picking colonies. All drug-resistant clones were picked and expanded in E8/Matrigel culture condition. A kill-curve of puromycin or G418 was also generated for the NSC lines using untargeted NSCs. Nucleofected NSCs were given 2–3 days to recover and resume proliferation. NSCs were then passaged 1:3 and given 24 hours to recover before they were exposed to 0.5μg/ml puromycin for 4–7 days (plus another 4–7 days selection by 100μg/ml G418 in the dual safe-harbor targeting case). To obtain clonal NSCs, 1~10 cells per 96-well were plated under standard NSC culture condition mentioned above. Surviving clones, which mostly were the only loosely clustered NSCs in the 96-well, were subsequently expanded.

### GFP rescue assay in HEK293T

GFP gene rescue assay in HEK293T cells was performed following similar procedure as previously described [2, 16]. First an AAVS1-EGIP* lentivector was constructed by inserting a 56bp fragment 5’–taaGTCCCCTCCACCCCACAGTGGGGCCACTAGGGACAGGATTGGTGACAGAAAAG–3’, which starts with a STOP codon and contains both AAVS1-TALEN target sequence (5’–CCCCTCCACCCCACAGTGGGGCCACTAGGGACAGGATTGGTGACAGAAA–3’) [3] and AAVS1-CRISPR/Cas target sequence (5’–GGGGCCACTAGGGACAGGAT–3’) [4], into enhanced GFP (EGFP) gene driven by constitutive EF1α promoter. The 293T-AAVS1-EGIP* cells were generated by transducing HEK293T with AAVS1-EGIP* lentivirus and selecting with 3μg/ml puromycin. To perform GFP rescue assay, 293T-AAVS1-EGIP* cells were plated at 2.5x10^5^ cells per well of a 12-well plate 1 day before lipofection. On the day of transfection, 1μg of tGFP donor (Addgene #26864) with various amounts of TALEN or CRISPR/Cas expression vectors was transfected using standard Lipofectamine 2000 protocol (Invitrogen). GFP+ cells were observed 48 to 72 hours post transfection and assessed by flow cytometry using Attune acoustic focusing cytometer (Life Technologies).

### NHEJ assay

For NHEJ assay, one million HEK293T cells were digested by 0.25% trypsin-EDTA, and collected by centrifugation at 150g for 5 minutes. The cells were nucleofected in 100 μL of 4D-Nucleofector SF Solution (Lonza, #V4XC-2024) containing a pair of pZT-CLYBL TALENs using 4D-Nucleofector System with program CM-130. The cells were cultured in DMEM (Life Technologies)/10% FBS (HyClone) for 3 days and collected. Genomic DNA (gDNA) from nucleofected HEK293T cells was isolated using DNeasy Blood and Tissue Kit (Qiagen) and used for PCR amplification of CLYBL TALEN cutting site. Primers are as follows: C13celF, 5’–CCTTCTTTGTTCTTCCCCAAG; C13celR, 5’–AAGATCACTTGAGCCCAGGA. PCR was performed using Phusion Hot Start II with reaction cycle parameters: 35 cycles of 98^o^C for 10 sec, 68^o^C for 15 sec, and 72^o^C for 30 sec, and a final extension for 5 min at 72^o^C. The PCR product was ~500 bps and purified using QIAquick PCR Purification Kit. To detect insertions/deletions (indels) caused by pZT-CLYBL TALENs, 200 ng amplicons were melted and randomly reannealed as follows: 95^o^C for 5 min, 95–85 ^o^C at −2^o^C/sec, 85–25^o^C at −0.1 ^o^C/sec, and then digested by 10 U of T7 endonuclease I (T7E1, New England BioLabs) at 37 ^o^C for 15 min. The digested sample was either subjected to electrophoresis on a 2% agarose gel or detected using the Agilent high-sensitivity DNA assay using the Agilent 2100 Bioanalyzer instrument. Cutting efficiency was calculated using formula: % gene modification = 100 × (1 − (1 − fraction cleaved)^1/2^). Alternatively, the PCR product was cloned into TOPO vector (Life Technologies) and ~50 clones were sequenced to directly calculate NHEJ efficiency without using T7E1.

### Antibody staining

Plated cells (iPSCs, NSCs, and differentiated progenies) were fixed in 4% paraformaldehyde for 15 minutes. Fixed cells were incubated in blocking buffer (1X PBS, 5% Normal Goat Serum, 0.3% Triton X-100) at room temp for 60 minutes, followed by an overnight incubation at 4°C with primary antibodies for TRA-1–60 (1μg/ml, mouse IgM, Millipore #MAB4360), NANOG (0.5μg/ml, rabbit IgG, PeproTech #500-P236), Smooth Muscle Actin 1 (1:400, Millipore, #CBL171), SOX17 (1:1000, R&D systems, #AF1924), and β3-tubulin (1:200, Cell Signaling Technology, #4466S), TUJ1 (1:1000, Sigma-Aldrich, #T8578), MAP2 (1:200, Life Technolgoies, #13–1500), GFAP (1:200, Life Technologies, #08–0063), diluted in antibody dilution buffer (1X PBS, 1% BSA, 0.3% Triton X-100). Cells were then incubated with secondary antibodies, Alexa Fluor 555 Goat Anti-Mouse IgM (Invitrogen #A-21426), Alexa Fluor 647 Goat Anti-mouse or rabbit IgG (Invitrogen #A-31634, #A-21244), Alexa Fluor 594 goat anti-mouse or rabbit IgG (Invitrogen #A11005, #A11012), or Tetramethylrhodamine rabbit-anti goat IgG (Invitrogen #A10532), diluted 500-fold in antibody dilution buffer for 2 hours at room temperature, protected from light. HaloTag live staining was performed using Oregon Green ligand and 15 min quick staining protocol as suggested by the manufacturer (Promega #G2801) Images were captured by Leica DMI3000B or AMG EVOS fluorescence microscopes and cameras, and processed by Adobe Photoshop.

### Luciferase assay and live imaging

Quantitative Nanoluc expression assay was carried out using defined numbers of cells counted by Cellometer Auto 2000 (Nexcelom Biosciences) and Nano-Glo Assay kit (Promega #N110) was administered following the manufacturer’s protocol. Results were analyzed using PekinElmer’s Victor X3 luminometer. Live Nanoluc staining was carried out by adding furimazine substrate from Nano-Glo kit directly into cell culture medium at 1:500 dilution and the images were taken by Olympus LV200 bioluminescence microscope.

### Southern blot

To verify homologous recombination at the *AAVS1* locus a 705-bp probe specific for both the endogenous PPP1R12C sequence and the 5’-homology arm of our constructs was synthesized by PCR amplification using primers AAV5Pb-F: 5’–GGCCTGGGTCACCTCTACG and AAV5Pb-R: 5’–GAACCAGAGCCACATTAACCG and DIG-dUTP labeling kit (Roche). 10μg of genomic DNA were digested with SphI overnight, after which Southern blotting and chemi-luminescence detection with CSPD were carried out following the instruction manuals of DIG High Prime DNA Labeling and Detection Starter Kit II (Roche). Based on the digestion pattern wild-type and targeted integration yield expected bands of 6.5kb and 3.8kb, respectively, due to the presence of an SphI site within our constructs. Verification of homologous recombination at the *CLYBL* locus was conducted similarly using a 528-bp probe specific for both the endogenous *CLYBL* sequence and the 5’-homology arm of our constructs synthesized using primers C13–5Pb-F: 5’–GGCATACCATCAAGTCCAAAG and C13–5pb-R: 5’–TTGGGGAAGAACAAAGAAGG. 10μg of genomic DNA were digested with AvrII overnight, which, after probe hybridization and imaging, yields expected bands of 5.4kb or 3.2kb for wild-type or targeted integrations for all *CLYBL* targeting donors, respectively. When BamHI was used with the *CLYBL* probe, a wild type band of 4.4kb and TI band of 11.2kb is expected for pC13N-CAGcopGFP.

## Results

### CLYBL–TALENs stimulate NHEJ and HDR in a novel safe-harbor like locus

**Figure 1:**
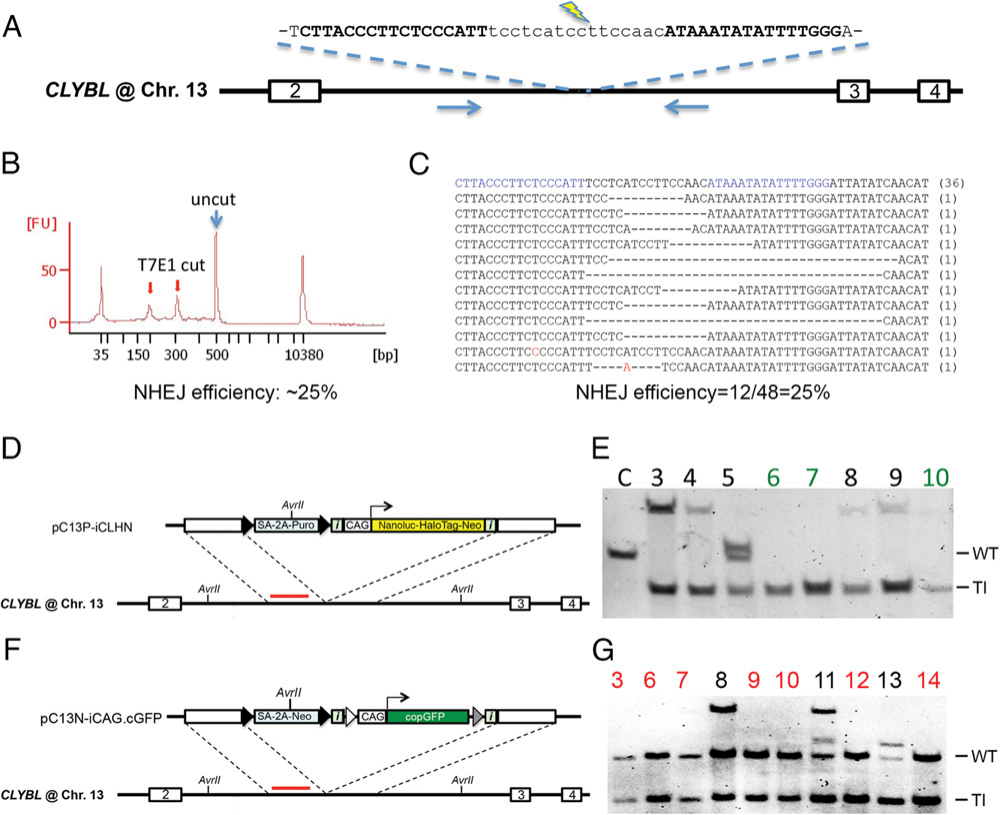
TALEN Targeting of a novel safe-harbor in the *CLYBL* gene. (A) Sequence of CLYBL-TALEN target site at intron 2 of *CLYBL*. Exon numbers are indicated in the boxes of genomic locus. Bold fonts indicate TALEN binding sequences: left CLYBL-TALEN binds 5’-CTTACCCTTCTCCCATT; right CLYBL-TALEN binds 5’-CCCAAAATATATTTAT. Blue arrows indicate the primers for NHEJ assay. (B) Capillary electrophoresis plot from T7E1 assay indicates that our CLYBL TALENs have ~25% NHEJ efficiency based on the calculation: % gene modification = 100 × (1 − (1 − fraction cleaved)1/2), where fraction cleaved = T7E1 cut/(T7E1cut+uncut). Red arrows indicate T7E1 cleaved bands, blue arrow indicates uncut PCR band. (C) CLYBL TALEN activity estimated by targeted amplicon sequencing confirmed 25% (12 alleles out of 48) gene editing efficiency. Blue fonts are TALEN binding sequence. (D and F) Schematics of gene targeting at *CLYBL* safe-harbor on Chr. 13. The donors shown have the same reporter cassettes as in Fig. 1C and 1E, including insulator-flanked CAG-copGFP or CAG-iCLHN, except that *CLYBL* homology arms were used and pC13N-iCAG.cGFP has neomycin resistant gene (Neo) instead of Puro. Red lines indicate CLYBL probe used with AvrII digestion in Southern blot analysis. (E and G) Southern blots of pC13P-iCLHN targeted (E) and pC13N-iCAG.cGFP targeted (G) NCRM5 iPSC clones. Because the same position of AvrII sites in both donors and the ability of CLYBL probe to recognize WT, TI and RI, WT band is 5.4kb due to two AvrII sites in intron 2, TI band is 3.2kb due to donor-introduced AvrII site, and any other additional bands are RI. Clones with 1TI-only are indicated in red numbers; clones with 2TI-only are indicated in green numbers. Clone “C” indicate the control untargeted cells.

We reasoned that using TALENs to target other safe-harbor like loci aside from the well-characterized AAVS1 site, which are usually actively transcribed regions, would avoid TALENs’ potential sensitivity to DNA methylation [17] and thus also enable efficient large transgene knock-in and sustained transgene expression. We chose to target the human *CLYBL* gene in Chromosome 13, which harbors a pseudosite for phiC31 random integration. Re-targeting of a pre-integrated R4 integrase site at *CLYBL* allowed for robust transgene expression in human embryonic stem cells and their differentiated lineages [14], likely because the *CLYBL* locus is transcriptionally active in almost all human cell types (www.biogps.org). These data suggested to us that *CLYBL* could potentially be utilized as a safe-harbor locus. To directly target the *CLYBL* locus in any human cells without pre-engineering, we set out to identify a unique TALEN target sequence at the *CLYBL* locus. To avoid the low specificity of NN RVD targeting G, we chose a TALEN target sequence composed of only A, C, or T, and identified one such sequence in intron 2 of *CLYBL*. The CLYBL TALENs (also referred as C13 TALENs) constructed using the pZT backbone showed 25% NHEJ gene editing efficiency in human HEK293T cells as measured by both T7E1 assay and targeted amplicon sequencing (Figs. 1A–1C). Using two donors that contain ~8kb cassettes expressing either copGFP or a Nanoluc-HaloTag fusion protein driven by the strong constitutive CAG promoter, we targeted the *CLYBL* locus with CLYBL-TALENs in human iPSCs (Figs.1D and 1F). Since *CLYBL* is actively expressed in human stem cells, puromycin or G418-selection was successfully used to enrich targeted cells expressing splicing acceptor-self-cleaving peptide (SA-2A)-linked drug-resistance genes. All drug-selected clones contained targeted integrations at the intended *CLYBL* locus and 38%-58% of the clones were TI-only without random integration (Fig. 1E, 1G, and Table 1). CLYBL-targeted human iPSC clones maintained stable reporter gene expression and normal karyotype, expressed pluripotency surface markers and were capable of differentiating into all three germ layers by *in vitro* embryoid body and *in vivo* teratoma assays (S1 Fig.).

**Table 1:** Targeted gene-addition efficiencies at *AAVS1* or *CLYBL* safe-harbor mediated by TALENs and various reporter donor vectors in human iPSCs.

**Locus**	**Donor**	**1TI-only (%)**	**2TI-only (%)**	**TI-only (%)**	**1TI+RI (%)**	**2TI+RI (%)**
*AAVS1*	iCAGcGFP	2/8 (25%)	2/8 (25%)	**4/8 (50%)**	1/8 (13%)	3/8 (37%)
*AAVS1*	iCLHN	5/14 (36%)	3/14 (21%)	**8/14 (57%)**	4/14 (29%)	2/14 (14%)
*AAVS1*	iCAGtdTom^a^	8/19 (42%)	7/19 (37%)	**15/19 (79%)**	3/19 (16%)	1/19 (5%)
*CLYBL*	iCAGcGFP	7/12 (58%)	0/12 (0%)	**7/12 (58%)**	5/12 (42%)	0/12 (0%)
*CLYBL*	iCLHN	0/8 (0%)	3/8 (38%)	**3/8 (38%)**	1/8 (12%)	4/8 (50%)
*CLYBL*	iCAGcGFP^a^	2/16 (12%)	6/16 (38%)	**8/16 (50%)**	0/16 (0%)	6/16 (38%)

TI = targeted integration; RI = random integration.

^a^Data from dual safe-harbor targeting experiments.

### Identical transgenes exhibit quantifiable, heightened expression in the *CLYBL* safe-harbor over *AAVS1*

Having displayed that the *CLYBYL* safe-harbor locus indeed enables persistent transgene expression throughout extended culture and *in vivo/in vitro* differentiation studies, we sought to further characterize the *CLYBL* locus by comparing it with the well-characterized AAVS1 safe-harbor locus. Using previously published AAVS1 TALENs[15] and the same donors as in *CLYBYL* targeting while swapping in homology arms specific for the AAVS1 site, we nucleofected, puromycin or G418 selected, and expanded iPSC clones harboring either the copGFP or the Nanoluc transgene at the AAVS1 safe-harbor. All surviving iPSC clones showed targeted integration (TI), with 50%–57% clones being TI-only without random integration (RI) (S2D and S2F Figs. and Table 1). These HDR efficiencies in human iPSCs are comparable to those reported using AAVS1-ZFNs or TALE13-based AAVS1-TALENs. We focused on TI-only clones and found they maintained pluripotency, normal karyotype, and reporter gene expression during extended iPSC culture and after teratoma formation (S3 Fig. and data not shown).

**Figure 2:**
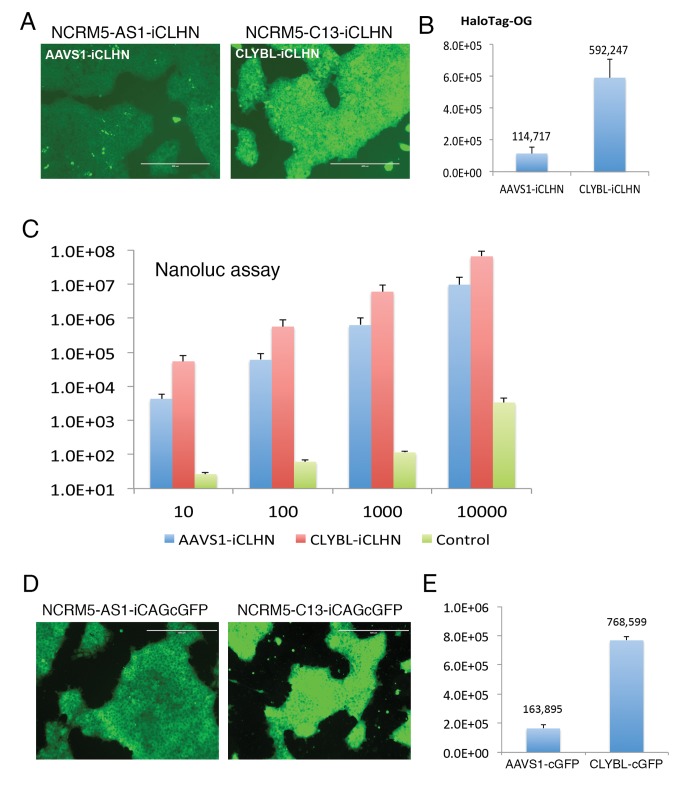
CLYBL safe-harbor enables heightened expression of transgenes. (A) Oregon green stained human iPSC clones with Nanoluc-HaloTag integrated in the *AAVS1* (AAVS1-iCLHN) or the *CLYBL* (CLYBL-iCLHN) safe-harbor bi-allelically without RI. Scale bar = 400 μm. (B) Comparison of HaloTag expression between *AAVS1* (AAVS1-iCLHN) and *CLYBL* (CLYBL-iCLHN) targeted NCRM5 iPSC clones using Oregon green (OG) ligand staining and flow cytometry. Y-axis = mean fluorescence intensity (MFI). Error bar = S.E.M. N = 3 (C) Nanoluc activity comparison of bi-allelically targeted AAVS1-iCLHN or CLYBL-iCLHN clones with untargeted NCRM5 control iPSCs. Y-axis is relative luciferase unit (RLU). X-axis is cell number. Data shown are the averages of 3 repeated measurements of three AAVS1-iCLHN clones, three CLYBL-iCLHN clones and one parental NCRM5 clone. Error bar = S.E.M. (E) Representative fluorescent images of CAG-driven copGFP expression in *AAVS1* (NCRM5-AS1-iCAGcGFP) or *CLYBL* (NCRM5-C13-iCAGcopGFP) targeted iPSC clones. Scale bar = 400 μm. (F) Quantitative comparison of copGFP expression between mono-allelically AAVS1 targeted (AAVS1-cGFP) and CLYBL targeted (CLYBL-cGFP) clones. Y-axis = MFI. Error bar = S.E.M. N = 2.

Having generated human iPSC clones containing identical reporter genes at either the *AAVS1* or *CLYBL* safe-harbor, we next sought to characterize the achievable levels of expression from either locus.We first investigated the effects of TI versus RI on quantification of reporter gene expression. Using copGFP-expressing AAVS1-targeted human iPSCs, we found that in clones where only correctly targeted integration occurred, the copGFP expression was both robust and proportional to the copy number of targeted integrations, with bi-allelic TI clones showing twice as much mean fluorescence intensity (MFI) as single TI clones (2TI = 316189 vs 1TI = 163895). Clones with additional RI showed larger variation of MFI compared to those without RI (1TI+RI = 199822±56239 vs 1TI = 163895±24859; 2TI+RI = 399528±62868 vs 2TI = 316189±19061), probably because some RIs resulted in additional fluorescence signal and some are silenced (S4 Fig.). Since random integration may add unpredicted transgene expression, we focused on TI-only clones and used identical reporter genes to compare the expression of transgenes integrated at AAVS1 and CLYBL loci. Using bi-allelic targeted human iPSC clones, we observed ~10-fold higher Nanoluc expression measured by relative luminescence unit (RLU) using a luminometer (RLU = 54177 vs 4314 from 10 cells, 567442 vs 59997 from 100 cells, 6008183 vs 626662 from 1000 cells) and ~5-folder higher HaloTag expression by Oregon Green ligand staining and flow cytometry (MFI = 592247 vs 114717) in *CLYBL* locus over the *AAVS1* locus (Figs.2A–2C). Furthermore, *CLYBL*-targeted and *AAVS1*-targeted clones exhibited >1000-fold and >100-fold higher expression over the negative control cells (RLU = 27 from 10 cells, 61 from 100cells, 115 from 1000 cells), respectively, suggesting that Nanoluc is a very sensitive reporter for detecting expression from 10 or potentially even fewer cells due to its extremely bright signal and low background. Using a different reporter gene but still focusing on TI-only clones, we also compared CAG-driven copGFP expression at mono-allelically targeted *AAVS1* and *CLYBL* loci, and found copGFP expression to be ~5-fold higher in *CLYBL* targeted iPSC clones (MFI = 768599) over *AAVS1* targeted clones (MFI = 163895) (Figs.2D–F).

### Dual knock-in of transgenes at *CLYBL* and *AAVS1* safe-harbors in human iPSCs enables rapid generation of stable reporter lines

**Figure 3:**
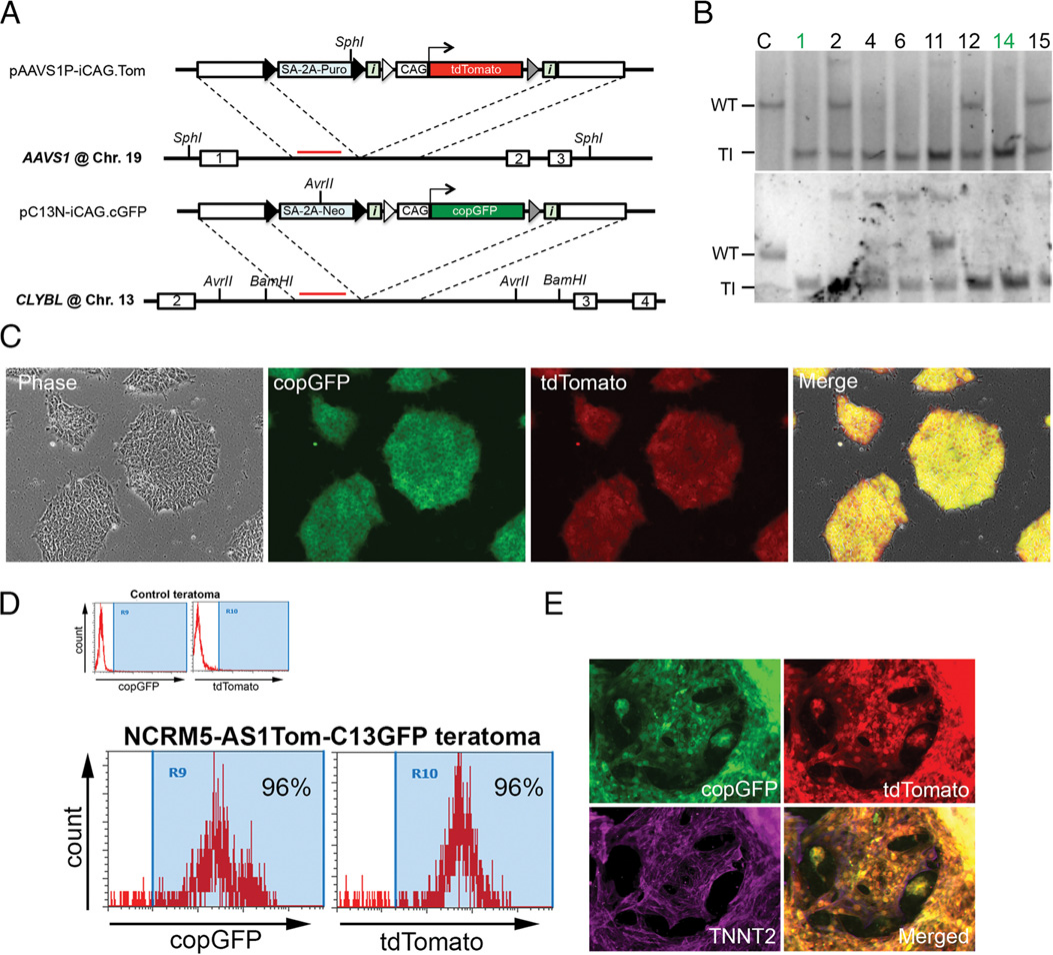
Double safe-harbor targeting in human iPSCs. (A) Schematic of dual safe-harbor gene addition of CAG-tdTomato in *AAVS1* and CAG-copGFP in *CLYBL*. The elements shown are similar to those in Figs. 1 and 2. Red bars indicate AAVS1 or CLYBL probes, used with SphI, AvrII or BamHI digestion in Southern blot analysis. (B) Southern blot of dual safe-harbor targeted iPSC clones. Top and bottom panels are results from AAVS1 probe/SphI digestion and CLYBL probe/AvrII digestion, respectively. The sizes of WT and TI bands were the same as shown in Fig. 1 and 2. Green numbers indicate clones with all four alleles targeted. (C) Phase contrast, copGFP and tdTomato fluorescent images of dual safe-harbor targeted iPSC NCRM5-AS1Tom-C13GFP clone 1. Merged image is from both fluorescent channels plus phase-contrast image. (D) After an 8-week *in vivo* differentiation, teratoma cells were isolated and still showed robust dual transgene expression by flow cytometry. (E) High-efficiency directed differentiation into beating cardiomyocytes expressing TNNT2 and both fluorescent proteins (also shown by online supplementary videos).

Encouraged by our highly active AAVS1 and CLYBL TALENs, we attempted to simultaneously target both safe-harbors by knocking-in two different donor constructs expressing either tdTomato or copGFP at the *AAVS1* or *CLYBL* locus, respectively (Fig. 3A). Surprisingly, the co-transfection efficiency of multiple donors was only slightly lower than that of a single donor (68% co-transfection vs 71%–79% single transfection, S5A Fig.) using nucleofection. We readily isolated dual-color clones after double drug-selection, and used Southern blot analysis to confirm 16 and 19 clones for CLYBL and AAVS1 targeting, respectively. 14/16 clones were confirmed to have both safe-harbors targeted, ranging from 1 allele for each safe-harbor to all 4 alleles for both. Among Southern confirmed clones, 79% were TI-only at AAVS1 locus, 50% were TI-only at CLYBL locus and 50% were TI-only at both loci (Fig. 3B, S5B and S5C Fig., Table 1), showing efficiencies comparable to that of single safe-harbor targeting. The double safe-harbor-targeted clones, including those with all four alleles modified, maintained normal karyotypes, iPSC morphologies and surface markers, and exhibited robust fluorescent protein expression with no observed silencing during extended culture (Fig. 3C–3D, S5D and S5E Fig.). They also efficiently differentiated into all three germ layers by *in vivo* teratoma formation and *in vitro* spontaneous embryoid body formation, and maintained transgene expression in teratoma as well as *in vitro* differentiated beating cardiomyocytes (Fig. 3D–3E, S5F and S5G Fig., and online video).

### TALEN-mediated dual safe-harbor loci targeting and stable transgene expression in human NSCs

**Figure 4:**
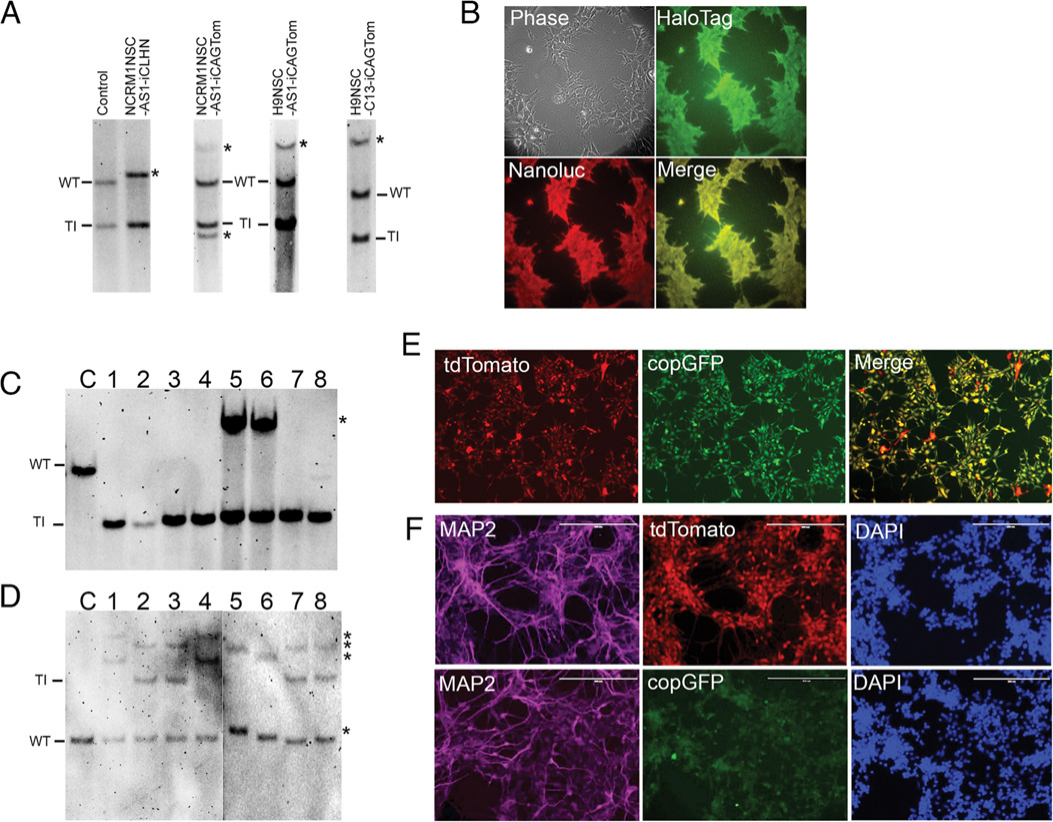
Safe-harbor targeting in human NSCs. (A) Southern analysis of Nanoluc-HaloTag (iCLHN) or tdTomato (iCAGTom) targeted NCRM1NSC or H9NSC at *AAVS1* or *CLYBL* locus using SphI or AvrII digestion, respectively. iPSCs with single TI of iCLHN at *AAVS1* allele were used as control. AAVS1 or CLYBL probes used to detect integrations at each safe-harbor are the same as shown in Fig. 4. Compared to the control, NCRM1NSC-AS1-iCLHN shows total loss of wild-type band (WT), indicating that all the cells in the polyclonal NSCs have bi-allelic TI at *AAVS1* locus. NCRM1NSCs or H9NSCs targeted by iCAGTom at *AAVS1* (AS1), and H9NSCs targeted by iCAGTom at *CLYBL* (C13) are estimated to have 44%-90% cells correctly targeted without random integrations (S6 Fig.). Asterisk indicates additional RI. (B) Phase, HaloTag fluorescence (stained by Oregon green) and Nanoluc luminescence (pseudo-colored red) images of NCRM1NSC-AS1-iCLHN showed ~100% targeted NSCs express Nanoluc-HaloTag. (C and D) Limiting dilution-derived clones of dual safe-harbor targeted NSCs, NCRM1NSC-AS1Tom-C13GFP, were confirmed by Southern using AAVS1 probe/SphI digestion and CLYBL probe/BamHI digestion as indicated in Fig. 4A. BamHI digestion generates a 11.2kb band due to TI at CLYBL, compared to 4.4kb for WT allele. Note that clone 2, 3, 7 and 8 have bi-allelic *AAVS1* TI and mono-allelic *CLYBL* TI plus an additional RI. Asterisk indicates additional RI. Lane C is control NCRM1NSC. (E and F) Fluorescent images of undifferentiated (E) and differentiated (F) NCRM1NSC-AS1Tom-C13GFP clone 3. MAP2+ committed neurons showed persistent tdTomato and copGFP expression. Nuclei were stained by DAPI. Scale bar = 400 μm.

The high efficiency of TALEN-mediated safe-harbor gene addition suggests that it is possible to directly target self-renewing somatic stem cells. Therefore, we chose to target pluripotent stem cell-derived, long term self-renewing neural stem cells (NSCs), which can proliferate and maintain their mulitpotency for more than 20 passages in *in vitro* culture [18]. We optimized our nucleofection protocol for NSCs and consistently achieved >70% transfection efficiency (S6A and S6B Fig.) using nucleofector 4D, program# DN-100, and 2ug total DNA/million NSCs. Using our AAVS1 and CLYBL TALENs with the donor vectors described in iPSC targeting, we first attempted reporter knock-in at single safe-harbor, *AAVS1* or *CLYBL*, in NCRM1 iPSC-derived NSC (NCRM1NSC) or H9 ESC-derived NSC (H9NSC). After drug selection we obtained polyclonal populations wherein reporter gene expression of either Nanoluc-HaloTag or tdTomato was displayed in nearly 100% of surviving NSCs. In NCRM1NSC-AS1-iCLHN cells, Southern blot analysis suggested that all the cells in the population had bi-allelic targeted insertion of Nanoluc-HaloTag at the *AAVS1* locus plus additional bi-allelic random integrations at another locus based on the absence of wild-type band and 1:1 ratio of TI:RI band intensity (Fig. 4A and S7A Fig.). They maintained robust Nanoluc and HaloTag expression during prolonged NSC expansion, are karyotypically normal, and can be induced to differentiate into Tuj1+ neurons and GFAP+ astrocytes (Figs.4B and E, S6C and S6D Fig.). Similarly, when the tdTomato reporter was targeted at either the *AAVS1* or the *CLYBL* locus in NCRM1NSCs or H9NSCs, at least 44% or 53% of NSCs were correctly targeted at *AAVS1* or *CLYBL* without random integration, i.e. TI-only, respectively. The rest of the polyclonal cells had only 1–2 random integrations (Fig. 4A and S7B-S7D Fig.). Our data indicated *AAVS1* or *CLYBL* targeting efficiencies in human NSCs are comparable to those in iPSCs. The tdTomato reporter gene integrated at the *CLYBL* locus also maintained robust expression in both undifferentiated NSCs and differentiated neurons (S6E and S6F Fig.). As it is unlikely to avoid a small fraction of RI within a polyclonal NSC population, a limiting dilution or counter-selection approach is needed to obtain clonal TI-only NSCs.

In light of our success performing dual safe-harbor gene addition in iPSCS, and because most drug-selected safe-harbor targeted iPSCs and NSCs we generated thus far had only targeted integrations, we attempted simultaneous dual safe-harbor targeting in NSCs and plated 1–10 NSCs per 96-well after drug selection. After 2-weeks, NSC clones were apparent in ~10% of the wells and those showing double fluorescent protein expression were expanded and analyzed. Southern blot analysis confirmed 4 out of 8 clones had *AAVS1* and *CLYBL* dual safe-harbor TI, plus 1 additional RI (Figs.4C and 4D). The dual safe-harbor targeted NSC clones maintained robust expression of both reporter genes during extended expansion under NSC culture conditions and after directed differentiation into committed neurons; no silencing of reporter genes at either safe-harbor was observed (Figs.4E-4F).

### Targeted integration of transgenes at safe-harbors has minimal effects on global and local gene expression in human iPSCs and NSCs

**Figure 5:**
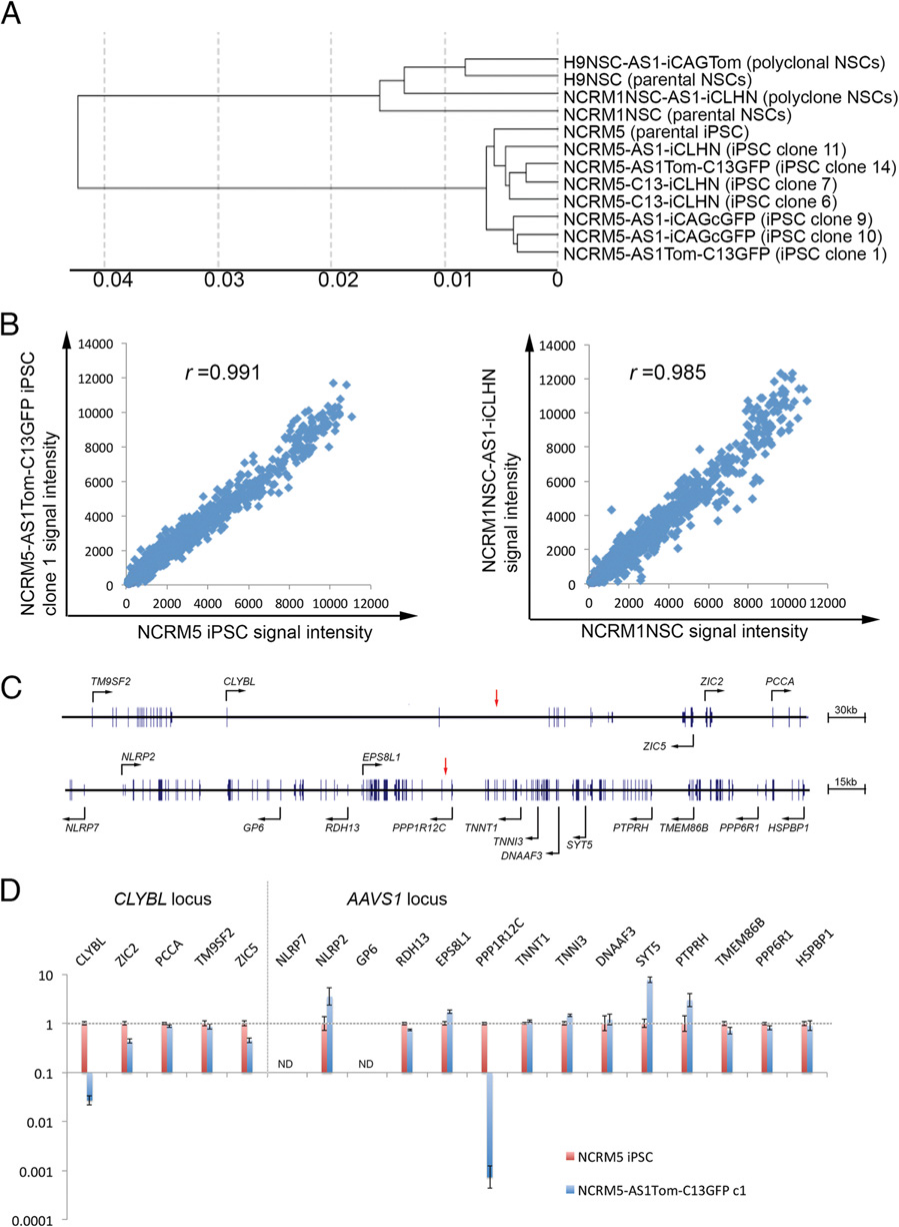
Effect of targeted integrations on global and local gene expression. (A) Dendrogram of non-targeted and safe-harbor targeted iPSCs and NSCs based on microarray analysis. The X-axis represents 1-*r*(correlation co-efficient). (GEO access# GSE55975) (B) Representatives of global gene expression profile comparison between untargeted and safe-harbor targeted cells. For NCRM5 iPSCs vs dual safe-harbor targeted NCRM5-AS1Tom-C13GFP clone 1, *r*=0.991. For NCRM1NSC vs AAVS1 targeted polyclonal NCRM1NSC-AS1-iCLHN. *r*=0.98. (C) Schematic of genomic region surrounding *CLYBL* and *AAVS1* safe-harbors. TALEN target sites are indicated by red arrows. A ~600kb region near the CLYBL-TALEN target site and a ~300kb region near the AAVS1-TALEN target site are depicted. (D) qRT-PCR analysis of the expression of genes near *AAVS1* and *CLYBL* safe-harbors, as shown in (C), in untargeted NCRM5 iPSCs and dual safe-harbor targeted clone 1. Values are normalized to the expression level of the β-actin (ACTB). ND = not detectable, due to Ct>35. Native *CLYBL* and *PPP1R12C* splicing transcripts were detected by specific primers recognizing exons downstream of TI (S2 Table). Fold-expression changes were calculated using the equation 2^-ΔΔCT^. Y-axis represents relative quantitation (RQ) in logarithmic scale. The error bars were calculated from 3 technical replicates and display the maximum (RQMax) and minimum (RQMin) expression levels that represent standard error of the mean expression level (RQ value). Collectively, the upper and lower limits defined the region of expression within which the true expression level value was likely to occur. The error bars were based on an RQMin/Max of the 95% confidence level.

The normal teratoma formation by mono- or bi-allelic safe-harbor engineered human iPSC confirmed that the genetic manipulations by our TALENs and donors did not drastically alter developmental potentials while exogenous reporters were stably expressed. To more clearly determine the effects the described genome engineering imposed on endogenous gene expression, we used microarray and quantitative RT-PCR (qRT-PCR) to compare global and local gene expression profiles, respectively, of non-targeted (parental) and mono- or bi-allelic safe-harbor targeted iPSCs and NSCs. The microarray results showed high correlation co-efficient between parental and targeted cells. The Pearson’s correlation co-efficient (*r*) between non-targeted iPSCs and single or dual safe-harbor targeted iPSC clones were 0.991~0.995. For NSCs, even though polyclonal targeted NSCs were used to compare with non-targeted lines, *r* was 0.985 between NCRM1NSC and NCRM1NSC-AS1-iCLHN and *r* was 0.991 between H9NSC and H9NSC-AS1-iCAGTom (Figs.5A and 5B). Interestingly, the lowest *r* we detected was associated with the cell line bearing the most random integrations, as every NCRM1NSC-AS1-iCLHN cell is estimated to have two additional random integrations (S7A Fig.). These results suggested that targeted integration of various transgenes at *AAVS1* and *CLYBL* safe-harbors in human iPSCs or NSCs, even at up to all four alleles, has minimal impact on genome-wide expression. In addition, we used qRT-PCR to analyze expression of neighboring genes near *CLYBL* locus in a >600kb region (Chr. 13: 100,108,918–100,795,018) and those close to *AAVS1* locus in a >300kb region (Chr. 19: 55,452,281–55,778,968) (Fig. 5C). To investigate how safely we could utilize both alleles of *CLYBL* and *AAVS1* in targeted integration, and thereby maximize the benefits of multiple genome engineering, we focused on comparing parental and dual safe-harbor targeted iPSC clones. There are 4 other genes within 600kb region surrounding the TI site in *CLYBL* and two of them (ZIC2, ZIC5) were down-regulated ~2-fold. In comparison, there are 13 other genes within 300kb region surrounding the TI site in *PPP1R12C* and three of them were up-regulated >2-fold (NLRP2 = 3.5-fold, SYT5 = 7.7-fold, PTPRH = 3.0-fold) (Fig. 5D). These observations suggested even with insulator elements at native *AAVS1* locus and added by transgene in our donor constructs, neighboring genes are more likely to be affected by TIs at the *AAVS1* locus as compared to the *CLYBL* locus. Because transgene integration in the introns of *CLYBL* and *PPP1R12C* can introduce deliberate splice site such as SA in our donors or cryptic ones found in some promoters [19] thus affecting normal splicing and transcription of entire endogenous gene, the changes in endogenous gene expression may not be detected by microarray probes or RT-PCR primers recognizing exons upstream of TI site. Therefore, we designed primers recognizing exons downstream of TI to detect native *CLYBL* and *PPP1R12C* transcripts (S2 Table). Consistent with a previous report [19], we observed in bi-allelic *AAVS1*-targeted cells *PPP1R12C* was downregulated >1000-fold. In contrast, endogenous *CLYBL* transcript only downregulated ~50-fold (Fig. 5D). We also performed qRT-PCR in other dual safe-harbor targeted iPSC and NSC clones and observed similar more significant downregulation of *PPP1R12C* than *CLYBL* (data not shown). In summary, the expression of neighboring genes of both safe-harbors appear minimally affected by the relatively large transgenes driven by strong CAG promoters we directed towards the intronic *PPP1R12C* and *CLYBY* safe-harbors, with endogenous *CLYBL* expression being less affected than *PPP1R12C*expression.

## Discussion

In the past two years, rapid and simple construction of TALEN and CRISPR/Cas designer nucleases has provided overwhelming options for genome engineering, as dozens of vectors are now available from the non-profit organization Addgene (www.addgene.org) and commercial companies. While designer nucleases’ activities and specificities are being improved, it is of great importance to investigate how best to safely and effectively apply these tools towards genetic modification in human stem cells, given that the products of these efforts will have broad impact on biomedical research and therapy. Our results demonstrate that safe-harbor TALEN-mediated HDR is a highly efficient method to generate targeted mini-gene transfer or reporter knock-in cell lines in both human iPSCs and NSCs.

We observed similar HDR efficiency among our TALENs, Goldy TALENs and CRISPR/Cas targeting the same sequence (S2 Fig.). These results suggest: 1) truncated N-/C- termini of TALENs from different *Xanthomonas* species exhibit comparable activities; 2) optimization of TALEN delivery parameters such as the TALEN/donor ratio is important to maximize the efficiency of TALEN-mediated gene editing, similar to what we have observed in ZFNs [2]; 3) for targeting transcriptionally active gene such as in GFP rescue assay in HEK293T, TALEN activity is not hampered by DNA methylation [20], and therefore can reach a high genome editing efficiency that is comparable to that of CRISPR/Cas. A recent report showed that specific TALEN design enabled similar or even higher genome editing efficiency than CRISPR/Cas [21], while another report suggested CRISPR/Cas is more efficient than TALEN in human iPSCs [5]. On the other hand, improvements of the specificities of both TALENs and CRISPR/Cas, such as NH RVDs for G [22, 23] and Cas9 nickase [24], have been reported recently. By only choosing RVDs that have high specificity and activity on A/C/T, we found no potential CLYBL-TALEN off-targets that can be predicted by a leading TALEN design program, TAL Effector Nucleotide Targeter (https://tale-nt.cac.cornell.edu). An additional search using TALENoffer program [25] provided a longer list of potential off-target sites that all differ from CLYBL-TALENs target sequence by 5 or more mismatches (data not shown). Such numerous mismatches in predicted off-target sites has been indicated to significantly reduce their specificity to, i.e., likelihood to be targeted by, the TALENs by >1000-fold in vitro and potentially even more in vivo [26]. While some report detailed the promiscuous nature of CRIPSR/Cas9 sequence recognition [27], recent studies showed the feasibility of obtaining gene-targeted human iPSCs with minimal off-target events by either CRISPR/Cas or TALEN technologies [28, 29, 30]. In addition, the use of Cas9 nickases has been shown to greatly diminish off-target activity [24].

Choosing to target introns inside of transcriptional units has the benefit of permitting selection strategies involving drug-selection genes driven by the endogenous, constitutively active *PPP1R12C* or *CLYBL* “safe-harbor” promoters. We repeatedly achieved 38%-79% correctly targeted knock-in of various reporter cassettes of up to 8 kb into either or both safe-harbors, ranging from single-allele TI to all four alleles simultaneously in human iPSCs (Table 1). Thus, we have generated fluorescent gene and Nanoluc luciferase reporter iPSC clones in which well-controlled gene expression provides sensitive and quantitative measurements of transcription (Fig. 2 and S4 Fig). The *CLYBL* safe-harbor on Chromosome 13 allows 5~10-fold stronger transgene expression than the *AAVS1* safe-harbor (Fig. 2), providing an alternative and potentially better solution for targeted gene transfer/knock-in and drug-screening, especially for weak promoter-driven transgenes.

In characterizing the effects of integrated transgenes on the endogenous targeted and neighboring genes, our findings were largely consistent with another recent publication that performed a detailed examination of three loci, two of which were either located in an intron of the dystrophin gene (DMD21) or only 3.8kb upstream of the 5’ end of the fucosyltransferase 8 gene (FUT8) [31]. In the described study, the extremely close proximity of the integration event to FUT8 had no effect on the endogenous gene’s expression levels. Likewise, the DMD21 intronic integration only marginally affected DMD21 expression levels, and this effect was only observed if the integration was in the same orientation as the DMD gene itself. In our current study, we investigated a >300kb region flanking the AAVS1-TALEN target site and a >600kb region around CLYBL-TALEN target site in bi-allelically targeted clones and found only *SYT5,* ~50kb downstream of *PPP1R12C,* was upregulated >5-fold while none in *CLYBL* region changed >5-fold (Fig. 5C-D). The small modulation of gene expression in our case may be due to the strength of the CAG promoter used to drive expression of our transgenes, as promoters have been found to affect transgene expression in a locus-specific fashion in a similar study in *AAVS1* or *CCR5* targeted B-lymphoblasts and hepatocyte cell lines [19]. Incorporation of SA in the targeted transgene reduced *PPP1R12C* transcription downstream of integration site by >1000-fold but only affected that of *CLYBL* by ~50-fold. Besides having few genes nearby, *CLYBL* is also well removed from any currently identified cancer-related genes based on a catalogue of somatic mutations in cancer (http://cancer.sanger.ac.uk/cancergenome/projects/census), with *ERCC5* being the closest at roughly 3000kb downstream. With minimal impact on global gene expression, higher integrated transgene expression level than *AAVS1*, the distance from any cancer-related genes, and the stability of transgene expression at this locus the *CLYBL* safe-harbor is a valuable tool for genome engineering purposes, especially when utilized in conjunction with other well-established safe-harbors. Further investigation of *CLYBL* safe-harbor designer nucleases and genetic modification would be of great interest.

Safe-harbor TALENs also enabled generation of engineered NSC populations and clones, which maintain multipotency and robust transgene expression during expansion and differentiation into neurons and astrocytes (Figs.4 and S6 Fig.). The frequencies of correctly targeted NSCs (44%-90%) are comparable to those of iPSCs (38%-79%), indicating that our safe-harbor TALENs are highly active in both stem cell types (Table 1 and S1 Table). One can expect that these safe-harbor TALENs will also stimulate genome editing in other somatic cells and stem cells, provided that the human cells have sufficient proliferation capacity and/or can be efficiently selected for targeted integration. With recombination-mediated cassette exchange (RMCE) elements [32] that are also present in some of our safe-harbor donor constructus, engineered “founder” iPSC and NSC lines can be further modified to create secondary cell lines without using TALENs (data not shown). The versatile toolbox of safe-harbor TALENs and donor vectors we have constructed provides a flexible system for engineering dual reporters and transgene cassettes in human iPSCs and NSCs.

## Supporting Information

S1 Fig Characterization of a human iPSC clone targeted at the *CLYBL* safe-harbor with a Nanoluc-HaloTag fusion protein reporter donor. (TIF)

S2 Fig Validation of pZT-AAVS1 TALENs in human HEK293T and their utility in genome engineering in iPSCs. (TIF)

S3 Fig Characterization of human iPSC clones targeted at the *AAVS1* safe-harbor. (TIF)

S4 Fig Flow-cytometric analysis of copGFP reporter expression in human iPSCs targeted at the *AAVS1* safe-harbor. (TIF)

S5 Fig Analysis of *AAVS1* and *CLYBYL* dual safe-harbor targeted human iPSC clones. (TIF)

S6 Fig Characterization of *AAVS1* and *CLYBYL* safe-harbor targeted NSCs. (TIF)

S7 Fig Quantification of targeted and random-integrations in polyclonal targeted NSC populations. (TIF)

S1 File Supplementary materials and methods. (DOCX)

S2 File Supplementary figure legends. (DOCX)

S1 Table Summary of safe-harbor targeted NSCs. (DOCX)

S2 Table Primers used in qRT-PCR analysis of *AAVS1* and *CLYBL* local gene expression. (DOCX)

S1 Video Beating cardiomyocytes differentiated from dual safe-harbor targeted human iPSCs (NCRM5-AS1Tom-C13GFP clone 1) showing tdTomato expression. (MOV)

S2 Video Beating cardiomyocytes differentiated from dual safe-harbor targeted human iPSCs (NCRM5-AS1Tom-C13GFP clone 1) showing copGFP expression. (MOV)
