# Supplementary figures and images for: Transcription Activator-Like Effector Nuclease (TALEN)-Mediated CLYBL Targeting Enables Enhanced Transgene Expression and One-Step Generation of Dual Reporter Human Induced Pluripotent Stem Cell (iPSC) and Neural Stem Cell (NSC) Lines
Source: PLoS One. 2015 Jan 14;10(1):e0116032. doi: 10.1371/journal.pone.0116032 (PMC4294658; doi:10.1371/journal.pone.0116032)

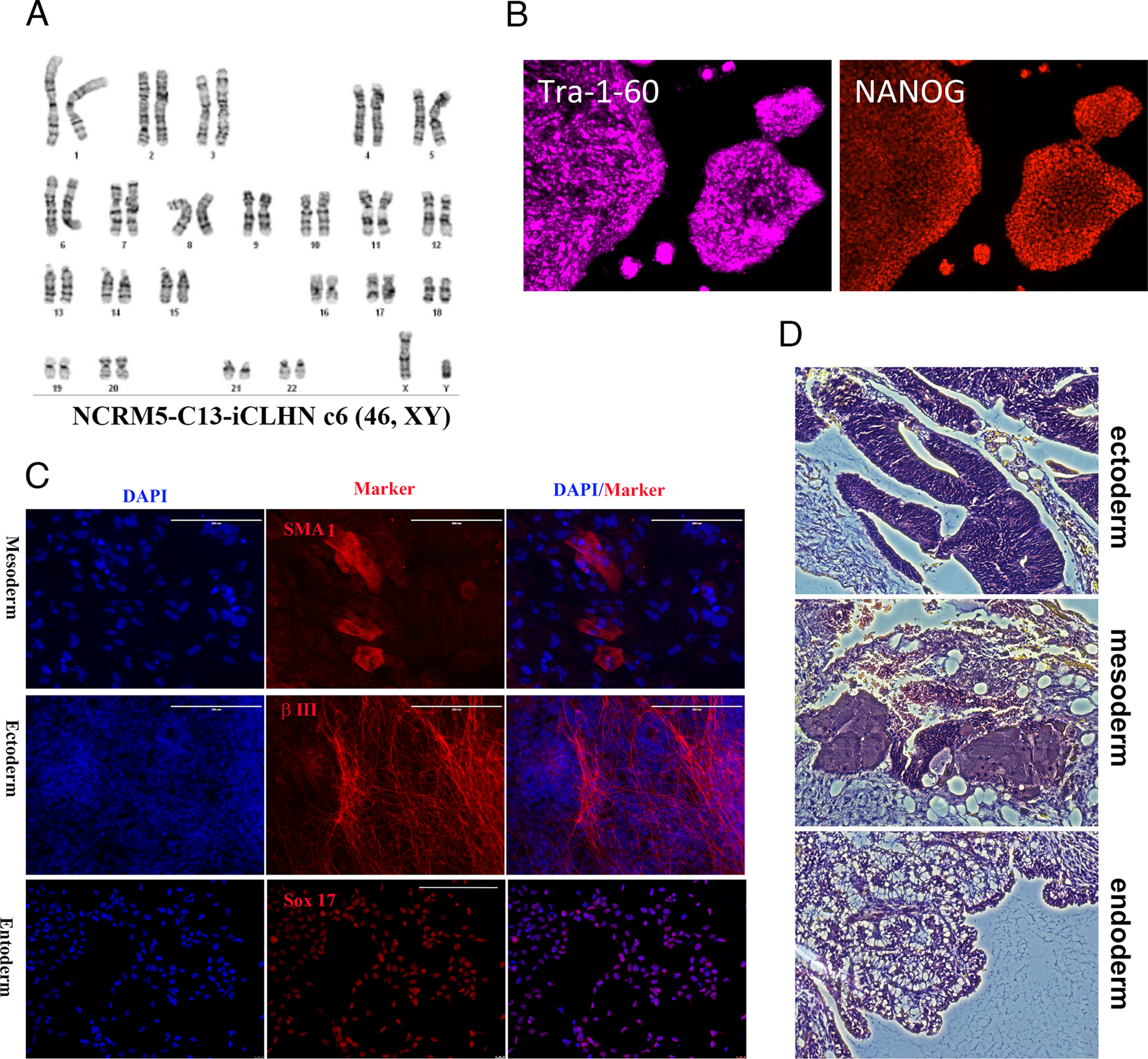

Supplement: S1 Fig — (TIF) [file pone.0116032.s001.tif]

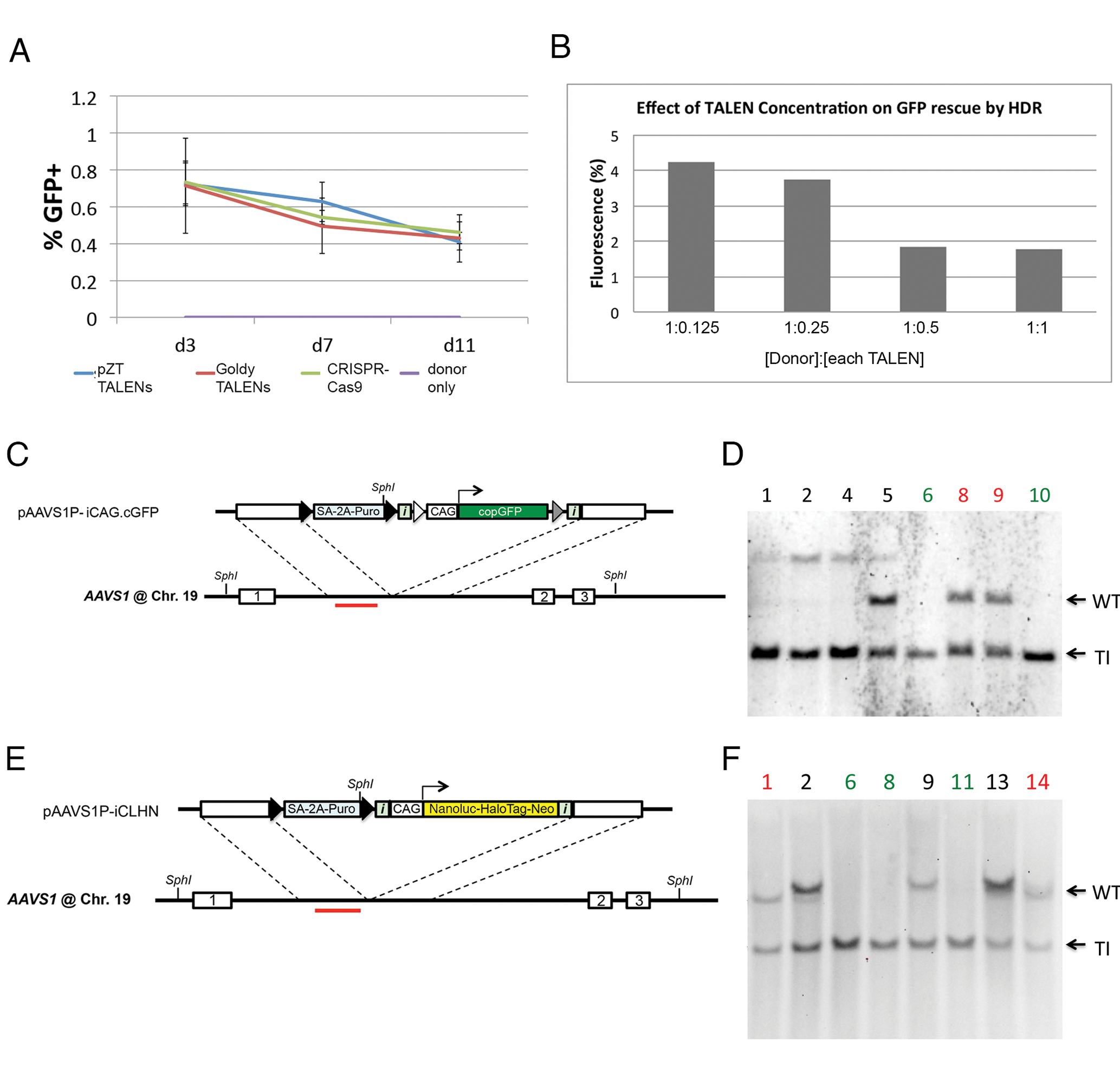

Supplement: S2 Fig — (TIF) [file pone.0116032.s002.tif]

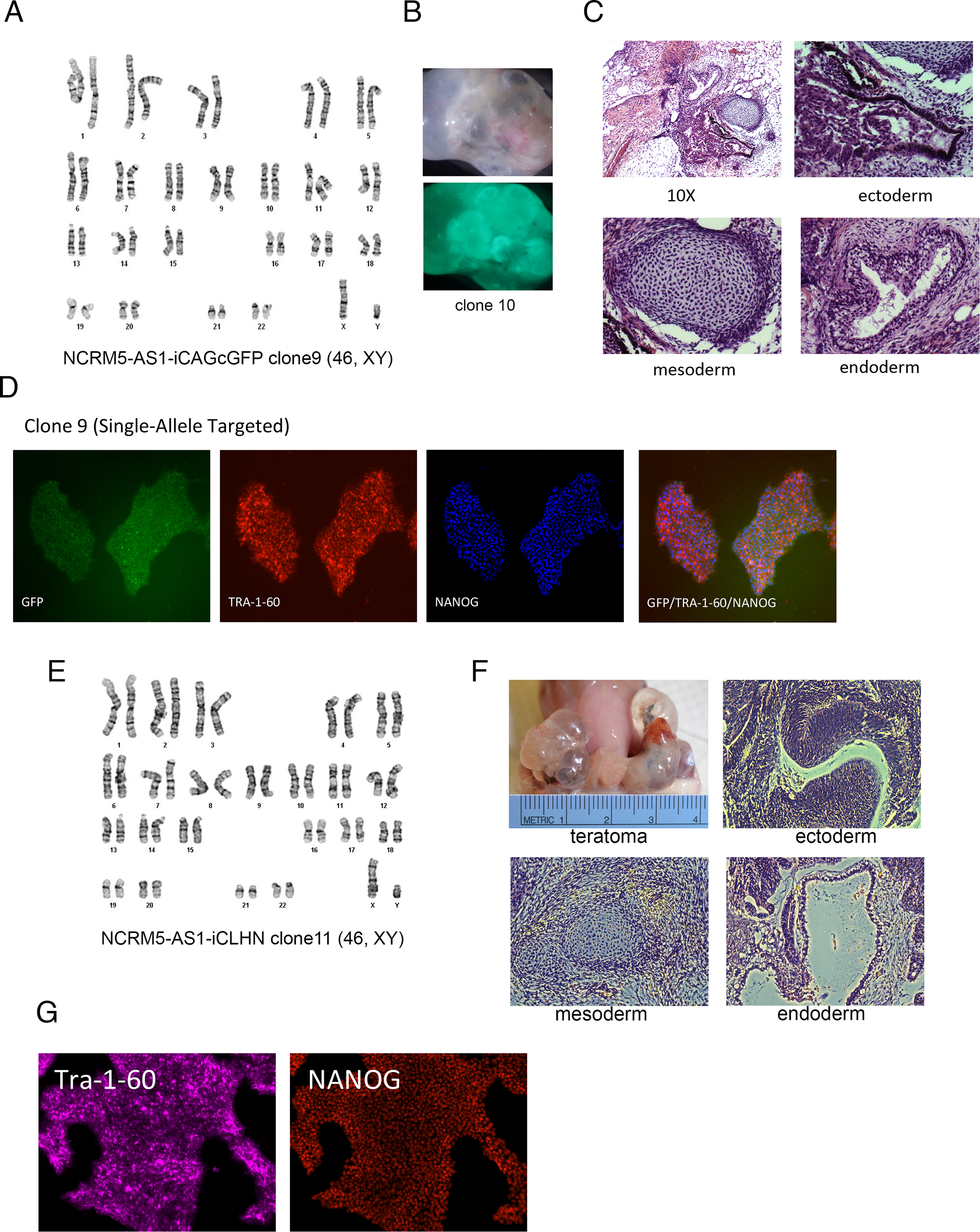

Supplement: S3 Fig — (TIF) [file pone.0116032.s003.tif]

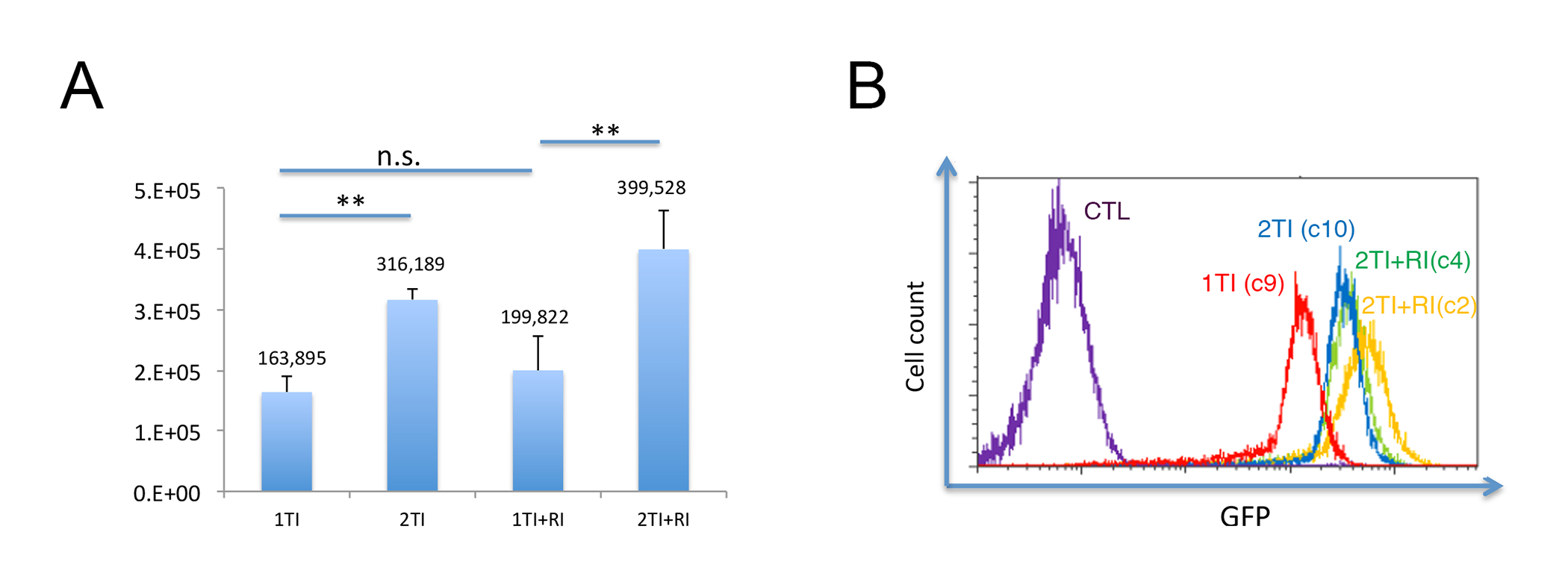

Supplement: S4 Fig — (TIF) [file pone.0116032.s004.tif]

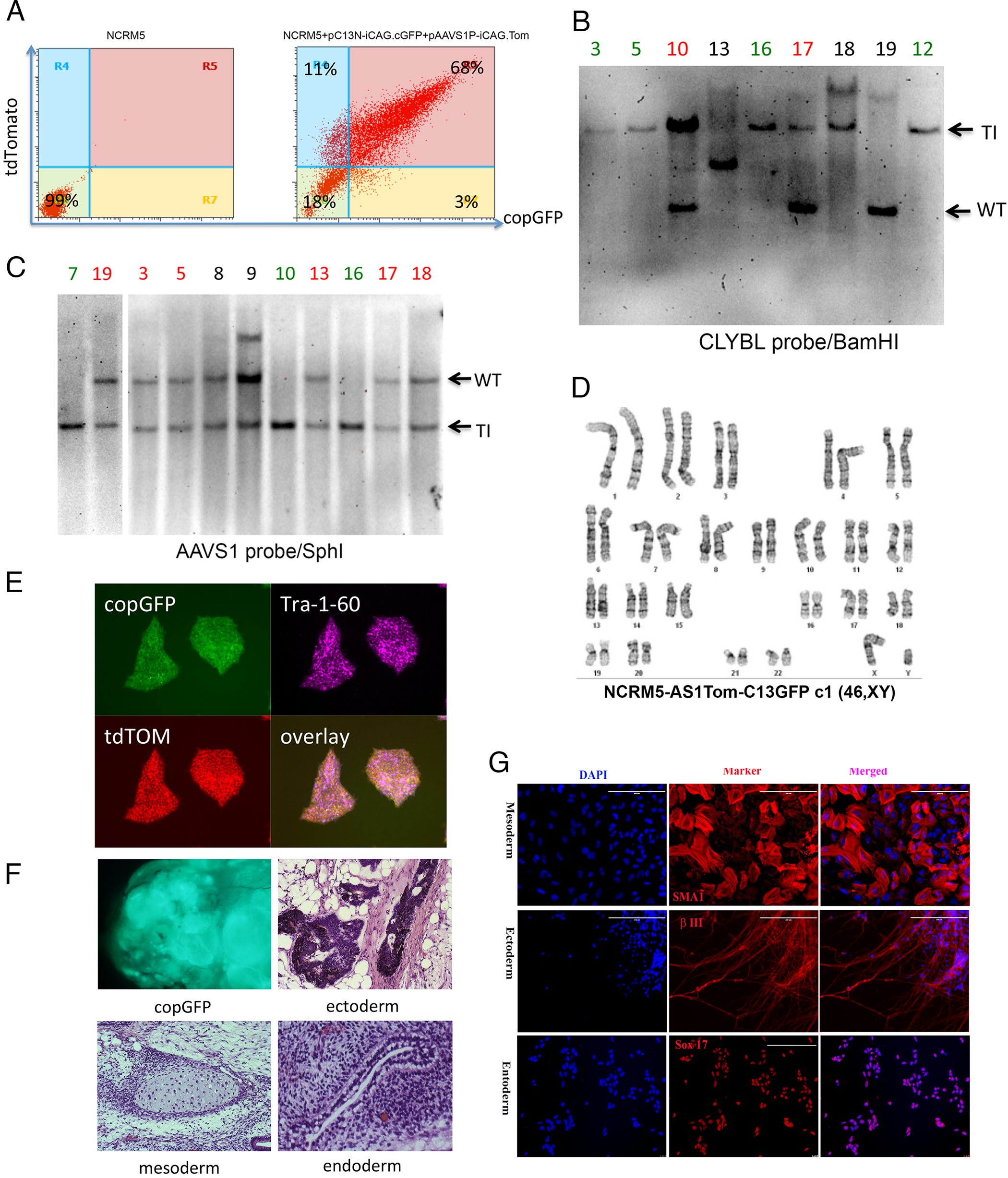

Supplement: S5 Fig — (TIF) [file pone.0116032.s005.tif]

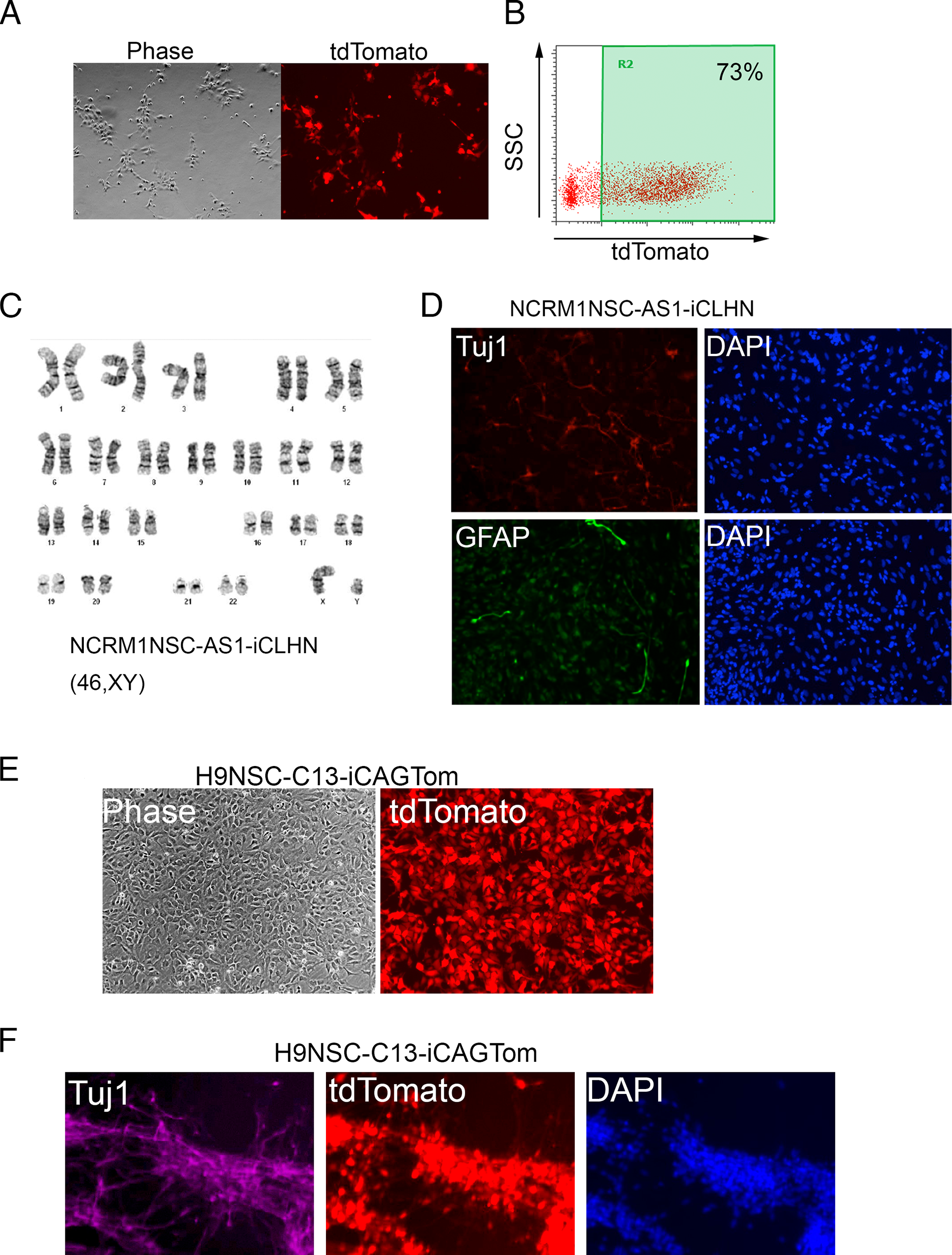

Supplement: S6 Fig — (TIF) [file pone.0116032.s006.tif]

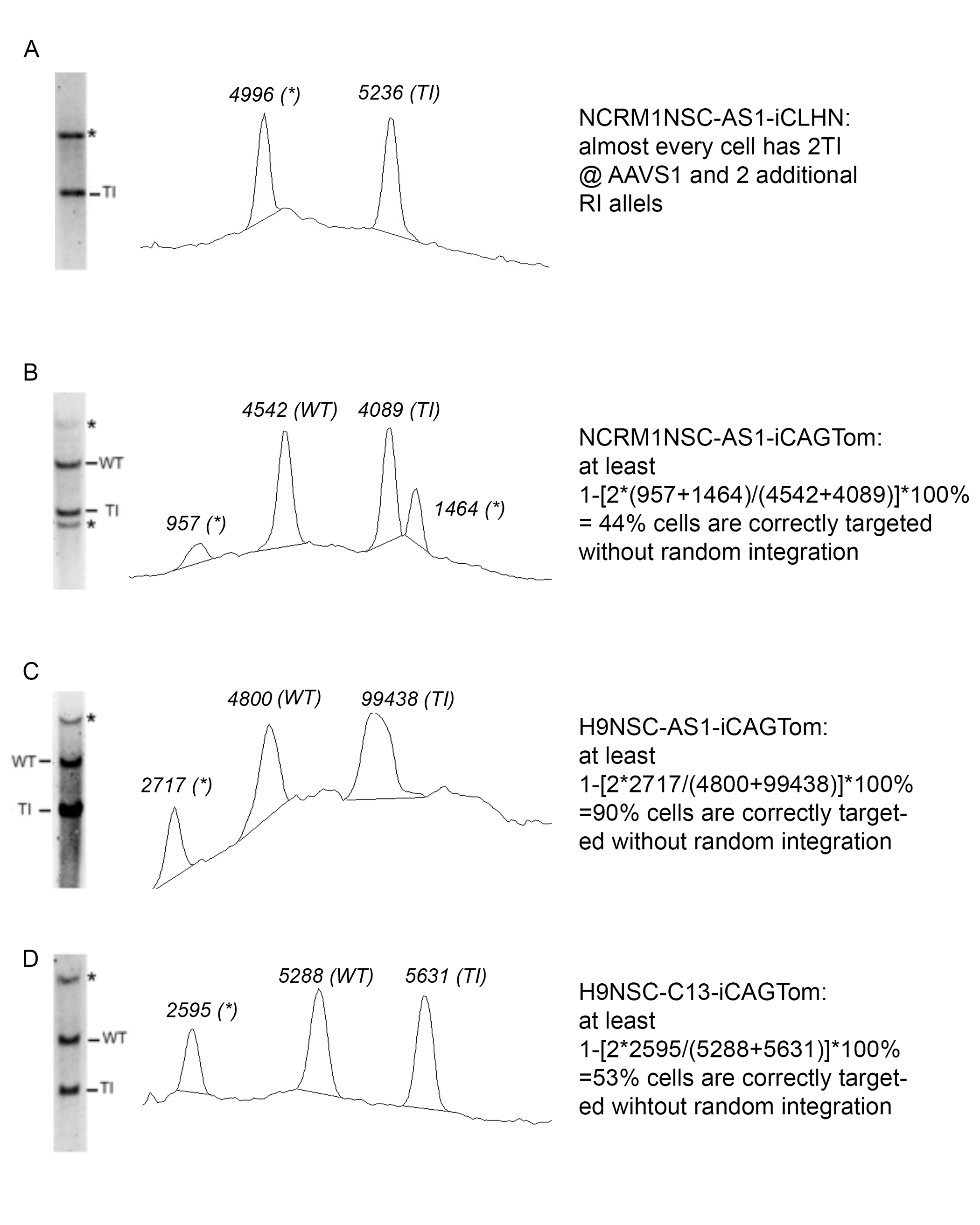

Supplement: S7 Fig — (TIF) [file pone.0116032.s007.tif]

**Table S1. Summary of safe-harbor targeted NSCs.**


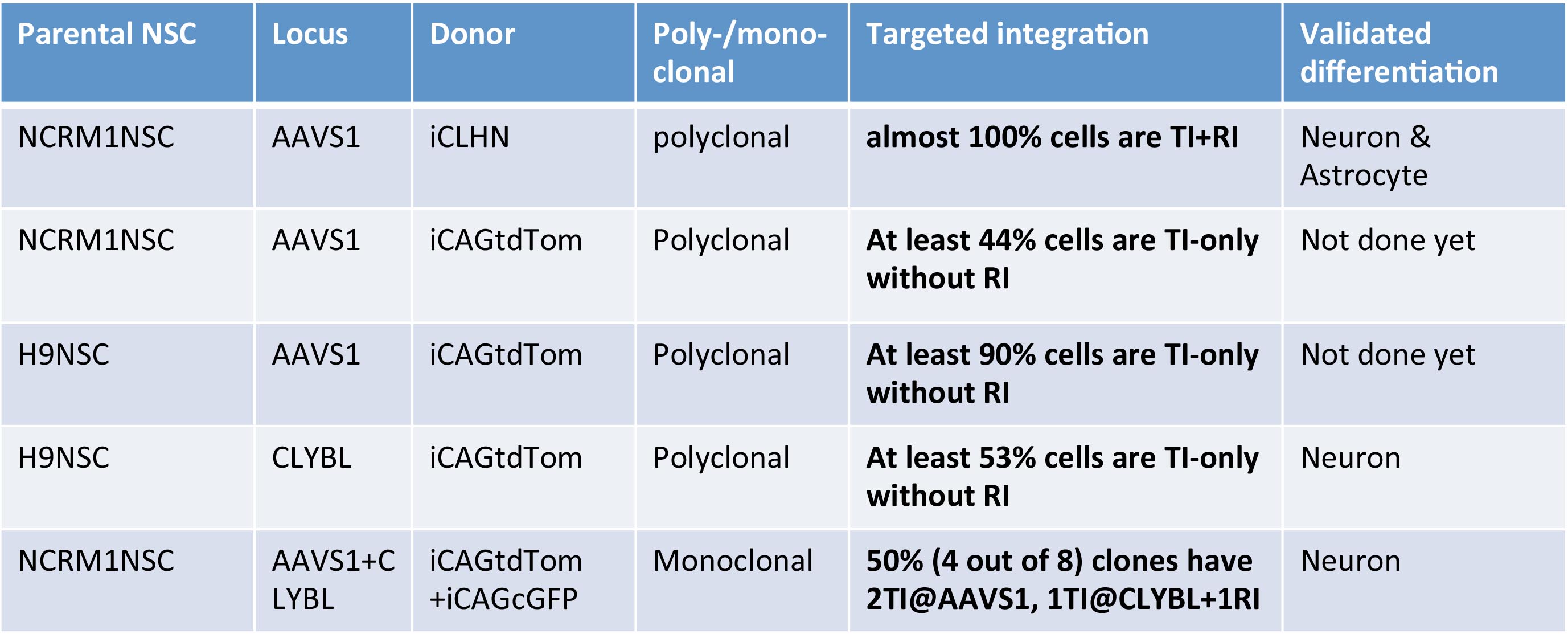

Supplement: S1 Table — (DOCX) [file pone.0116032.s010.docx]
