# Supplementary material for: Transcription Activator-Like Effector Nuclease (TALEN)-Mediated CLYBL Targeting Enables Enhanced Transgene Expression and One-Step Generation of Dual Reporter Human Induced Pluripotent Stem Cell (iPSC) and Neural Stem Cell (NSC) Lines
Source: PLoS One. 2015 Jan 14;10(1):e0116032. doi: 10.1371/journal.pone.0116032 (PMC4294658; doi:10.1371/journal.pone.0116032)
Supplement: S1 File — (DOCX) [file pone.0116032.s008.docx]

**Supplementary Materials and Methods**

**In vitro iPSC differentiation**

For *in vitro* spontaneous three germ layer differentiation, the undifferentiated targeted colonies were grown to 70 – 80% confluent in Essential E8™ Medium (Invitrogen), dissociated by Dispase® solution (2 mg/mL, Invitrogen), and subcultured in ultra-low attachment dishes(Corning) to generate EBs with Essential E6™ Medium ((Invitrogen) for 6 days. On day 7, EBs were seeded on Geltrex-coated plates for an additional 10 to 14 days to allow differentiation into three germ layer lineages in the same Essential E6™ Medium. The cells were then subject to immunocytochemistry analysis.

For direct differentiation into definitive endoderm, the undifferentiated targeted iPSCs were first adapted from Essential E8™ Medium to mTeSR™culture medium (StemCell™ Technologies) for one passage and then subject to induction using STEMdiff™ definitive endoderm kit (StemCell™ Technologies) for 4 to 5 days, following manufacturer’s protocol. Formation of definitive endoderm was evaluated by immunofluorescence staining of Sox 17.

For directed differentiation into cardiomyocytes (CM), we followed a published protocol using GSK3 inhibitor (CHIR99021) and Wnt signaling inhibitor (IWP2)[[1](#_ENREF_1)]. The live beating CM video was taken by Leica DMI6000 microscope and processed by ImageJ software.

**Teratoma formation and assay**

The hiPSCs were cultured to 80-90% confluency and disassociated with collagenase IV. The cells (2x10^6^) were re-suspended in PBS and then mixed with 50% volume of Matrigel, and then injected subcutaneously into immunocompromised NSG mice. Visible tumors were harvested 6-7 weeks after injection. Half of each tumor was digested with 0.25% trpsin-EDTA (Invitrogen) for flow cytometry analysis of EGFP expression. The remaining half was fixed in 4% paraformaldhyde, paraffin sectioned, and stained with haematoxylin and eosin for histological analysis.

**In vitro NSC differentiation**

Targeted NSCs were differentiated to Tuj1- and MAP2-expressing neurons using DMEM/F12 (Life Technologies, #10565-042), 1x GlutaMAX (Life Technologies, #35050-061), 2% StemPro hESC supplement (Life Technologies, #A1000701), 1.8% BSA, BDNF (20ng/ml, R&D, #248-BD), and GDNF (20ng/ml, R&D, #212-GD) for two weeks. Targeted NSCs were differentiated to GFAP-expressing astrocytes using BMP2/CTNF Differentiation Medium consisting of DMEM/F12, 1XGlutaMax, 1X NEAA, 1% N-2 supplement and 1% FBS for 35 days, following Gibco Neurobiology Protocol (<https://www.lifetechnologies.com/us/en/home/references/protocols/neurobiology/neurobiology-protocols/differentiating-neural-stem-cells-into-neurons-and-glial-cells.html>).

**Microarray hybridization and analysis**

Approximately one million pelleted cells were sent to Expression Analysis (Durham, NC USA) for extraction, amplification, labeling and hybridization of RNA to an Illumina (San Diego, CA USA) HT-12 v4 BeadChip array (Illumina, #BD-103-0204). The Gene Expression module of the Illumina GenomeStudio software package was used to process the array image files, normalize data with background subtraction, and generate scatter plots and dendrograms.  The data were then exported to Excel for further “cleaning” by removing any probes in which the intensity value was <50 for all samples and all intensity values less than 1 were converted to 1.  This “cleaned” file was used for further analysis of the microarray dataset. The microarray dataset can be accessed at: <http://www.ncbi.nlm.nih.gov/geo/query/acc.cgi?acc=GSE55975>

**qRT-PCR analysis**

Total RNA from cells was extracted using TRIzol® reagent (Life Technologies). Reverse transcription for complementary DNA (cDNA) synthesis was performed using SuperScript® III First-Strand Synthesis System (Life Technologies) and real-time polymerase chain reaction (PCR) was performed with SYBR Green technology and using primers listed in **Table S2** on Applied Biosystems ViiA7 (Life Technologies).

**Estimation of percentage of correctly targeted NSCs in the polyclonal population**

For polyclonal targeted NSCs, we used the following methods and Southern results to estimate the percentage of cells that are correctly targeted: % (correctly targeted cells) = % (cells without RI) = 1- % (cells with RI). On polyclonal NSC Southern blots, each RI band represents at least one RI allele (1RI). If there is only 1RI for every cell with RI, the % (cells of RI) = 2X [(volume of RI bands) / (total volume of WT and TI bands)] because the total volume of WT and TI bands represents two alleles in each cell. When there are ≥1RI in every cell with RI, the % (cells of RI) ≤ 2X [(volume of RI bands) / (total volume of WT and TI bands)]. Therefore, using the volume of each Southern band to calculate, %(correctly targeted cells) ≥ 1-[2xRI/(WT+TI)] x 100%.

**Reference:**

1. Lian X, Zhang J, Azarin SM, Zhu K, Hazeltine LB, et al. (2013) Directed cardiomyocyte differentiation from human pluripotent stem cells by modulating Wnt/beta-catenin signaling under fully defined conditions. Nature protocols 8: 162-175.
