# Supplementary material for: Transcription Activator-Like Effector Nuclease (TALEN)-Mediated CLYBL Targeting Enables Enhanced Transgene Expression and One-Step Generation of Dual Reporter Human Induced Pluripotent Stem Cell (iPSC) and Neural Stem Cell (NSC) Lines
Source: PLoS One. 2015 Jan 14;10(1):e0116032. doi: 10.1371/journal.pone.0116032 (PMC4294658; doi:10.1371/journal.pone.0116032)
Supplement: S2 File — (DOCX) [file pone.0116032.s009.docx]

**Supplementary Figure Legends**

**Figure S1: Characterization of a human iPSC clone targeted at the CLYBL safe-harbor with a Nanoluc-HaloTag fusion protein reporter donor.**

(**A-D**) NCRM5-C13-iCLHN clone 6 shows normal karyotype (**A**), stains positive for the pluripotency markers TRA-1-60 and NANOG (**B**), and is capable of *in vitro* (**C**) and *in vivo* (**D**) differentiation into three germ layers.

**Figure S2. Validation of pZT-AAVS1 TALENs in human HEK293T and their utility in genome engineering in iPSCs**

(**A**) Time course of percentages of GFP+ cells gene-corrected by pZT-AAVS1-TALEN, Goldy AAVS1-TALEN or AAVS1-CRISPR/Cas in 293T-AAVS1-EGIP* cells suggested they have similar genome editing efficiency. (**B**) Ratio of donor:TALEN affects gene targeting efficiency in HEK293T cells. Different ratios were compared using pZT-AAVS1 TALENs and GFP rescue assay at day 3 after transfection. (**C** and **E**) Schematics of reporter genes knock-in at *AAVS1* safe-harbor. The targeting donors, which contain a splicing acceptor (SA)-linked 2A self-cleaving peptide sequence, puromycin selection gene (Puro), loxP (black triangle), lox2272(white triangle), lox511 (grey triangle), and insulator (i) elements-flanked CAG promoter-driving copGFP (**C**) or the Nanoluc-HaloTag fusion protein (**E**), are inserted into an AAVS1-TALEN target sequence in intron 1 of the *PPP1R12C* gene. Exon numbers are indicated in the boxes of genomic locus. Dotted lines indicate homology arms used in the gene targeting. Red lines indicate AAVS1 probe used with SphI digestion in Southern blot analysis. (**D** and **F**) Southern blots of pAAVS1P-iCAG.cGFP targeted (**D**) and pAAVS1P-iCHLN targeted (**F**) NCRM5 iPSC clones. Because the same position of SphI sites in both donors and the ability of AAVS1 probe to recognize wild-type (WT), targeted integration (TI) and random integration (RI), WT band is 6.5kb due to two SphI sites flanking exon 1 and exon 3, TI band is 3.8kb due to donor-introduced SphI site, and any other additional bands are RI. Clones with 1TI-only are indicated in red numbers; clones with 2TI-only are indicated in green numbers.

**Figure S3: Characterization of human iPSC clones targeted at the AAVS1 safe-harbor.**

(**A**) Normal karyotype of NCRM5-AS1-iCAGcGFP clone 9 (**B-C**) Teratoma derived from NCRM5-AS1-iCAGcGFP clone 10 showed persistent copGFP expression and three germ layer differentiation capacity. (**D**) Pluripotent surface marker staining of undifferentiated NCRM5-AS1-iCAGcGFP clone 9. (**E**) Normal karyotype of NCRM5-AS1-iCLHN clone 11. (**F-G**) NCRM5-AS1-iCLHN clone 11 forms teratoma with three germ layer lineages by *in vivo* differentiation (**F**) and stains positive for pluripotency markers in iPSC culture (**G**).

**Figure S4: Flow-cytometric analysis of copGFP reporter expression in human iPSCs targeted at the AAVS1 safe-harbor.**

(**A**) Summary of GFP mean fluorescent intensity (MFI) in clones with only single TI (1TI), only double TI (2TI), and additional RI (1TI+RI or 2TI+RI). Comparisons show 2TI clones have significantly higher expression than 1TI clones (p<0.01, **) with nearly double MFI (Y-axis). Additional RI does not significantly (n.s.) increase expression level (1TI+RI vs. 1TI, p>0.1). CAG-copGFP and CAG-EGFP clones (Luo et al., unpublished data) were grouped together based on integration profile. N>=3. Error bar=S.E.M. (**B**) Flow cytometry histogram of NCRM5-AS1-iCAGcGFP iPSC clones, showing difference of fluorescence intensities among 1TI (c9), 2TI (C10), and 2TI+RI clones (c2, c4). Note that some clones with additional RI resulted in elevated MFI (c2) and some not (c4).

**Figure S5: Analysis of AAVS1 and CLYBYL dual safe-harbor targeted human iPSC clones.**

(**A**) Co-nucleofection efficiency of two-color targeting donors (right plot) compared to the negative control (left plot). (**B**) Clone gDNA were digested by BamHI and analyzed using CLYBL probe. WT=4.4kb, TI=11.2kb. (**C**) Clone gDNA were digested by SphI and analyzed using AAVS1 Probe. WT=6.5kb, TI=3.8kb. Clone numbers are on the top of the blots, Red=1TI, Green=2TI. Targeting schematic was shown in Figure 4. (**D**) Normal karyotype of a double-targeted clone (**E**) Pluripotent surface marker TRA-1-60 is co-expressed with both tdTomato and copGFP. (**F**) Fluorescent microscopy (only GFP channel is shown) and H&E staining of teratoma, showing three germ layer lineages. (**G**) In vitro differentiation into three germ layer lineages.

**Figure S6: Characterization of AAVS1 and CLYBYL safe-harbor targeted NSCs.**

(**A**) Representative phase and fluorescent images of CAG-tdTomato donor transfected NSCs at day2 after nucleofection. 2μg DNA/million cells were used. (**B**) Representative flow cytometry plot of transfected NSCs. (**C**) normal karyotype of AAVS1-targeted polyclonal NCRM1NSC-AS1-iCLHN. (**D**) NCRM1NSC-AS1-iCLHN can differentiate into TUJ11+ neurons and GFAP+ astrocytes. (**E**) pC13N-iCAG.Tom targeted H9NSCs show nearly 100% strong tdTomato expression. (**F**) TUJ1+ neurons differentiated from H9NSC-C13-iCAGTom show robust tdTomato expression.

**Figure S7: Quantification of targeted and random-integrations in polyclonal targeted NSC populations, related to Figure 5.**

(**A**) Since there is no WT allele band and RI band has similar volume to TI band which represents 2 alleles in the genome, every cell is estimated to have 2TI at AAVS1 locus and additional 2RI. (**B-D**). Percentages of correctly targeted cells in the safe-harbor targeted NSC polyclonal population were estimated using the formula %(correctly targeted cells) ≥ 1-[2xRI/(WT+TI)] x 100% described in Supplementary Experimental Procedures.
