# Supplementary material for: Transcription Activator-Like Effector Nuclease (TALEN)-Mediated CLYBL Targeting Enables Enhanced Transgene Expression and One-Step Generation of Dual Reporter Human Induced Pluripotent Stem Cell (iPSC) and Neural Stem Cell (NSC) Lines
Source: PLoS One. 2015 Jan 14;10(1):e0116032. doi: 10.1371/journal.pone.0116032 (PMC4294658; doi:10.1371/journal.pone.0116032)
Supplement: S2 Table — (DOCX) [file pone.0116032.s011.docx]

**Table S2. Primers used in qRT-PCR analysis of AAVS1 and CLYBL local gene expression.**

| **Gene** | **Forward Primer** | **Reverse Primer** |
| --- | --- | --- |
| NLRP7 | AGCTCGTGGATTGTGGATTC | CCAACAGCTTCTTGATTTCCA |
| NLRP2 | TCAAAACAGAAGCACAAGCGTT | CAGCTCTTCCACATCTCCCG |
| GP6 | AGCTTGTGGTCACAGGAACC | ATGAGACGGTCAGTTCAGCG |
| RHD13 | CTCAATCACCATGTCAACGC | AGAATGTCCACTCGCTCCTC |
| EPS8L1 | GGACCCAGTTGAGAAACAGC | GCTCAGATTCTGGCTCCAAG |
| PPP1R12C | ATGGAGGGGCTGCTGAAG | CTCGTGTCATGAAGGAGCAA |
| TNNT1 | GGCAGAGCCAGAAGAGGAAC | GATGTCATCGAAGTCAACGC |
| TNNI3 | GTGAAGAAGGAGGACACCGA | CAGTAGGCAGGAAGGCTCAG |
| DNAAF3 | AACGTGACGGAGCTGCTC | AGGAAGTGGACGGTGAAAGA |
| SYT5 | CGGAAGAAGAAAACCACCAT | CAGCTCCACCTGCACCTT |
| PTPRH | CAGAACTCAGGGATCTGGTCT | GGGAGAGTTGCTGGTACTTG |
| TMEM86B | CTTGTGTGCTCGGCTGTG | AAGGCCCAGACGTAGAGGA |
| PPP6R1 | AGAGGAGGACGAGGAAGAGG | ACTGTCTGTGCTGCCTCCA |
| HSPBP1 | GTGCAGTATCCCGCCAGATG | CGATTCTTGAGGGTCTCCCC |
| CLYBL | AAGACATTGATCTGGGCCCT | ATCAGGCTGGAAGGAAGGAC |
| ZIC2 | AAGATCCACAAAAGGACCCA | TGCATGTGCTTCTTCCTGTC |
| PCCA | CGAGAAGCAGGTGGAAACAT | AATTTGTTCAATTCTGCGGC |
| TM9SF2 | AAAAAGAGCGACGAGTGCAA | CTTGGCAAAAATCAAACGCT |
| ZIC5 | ACCTCAAGATCCACAAGCGT | GGGAATGTTTCTTCCGATCA |
| ACTB | CCTTGCACATGCCGGAG | GCACAGAGCCTCGCCTT |
